# Contrastive self-supervised clustering of scRNA-seq data

**DOI:** 10.1186/s12859-021-04210-8

**Published:** 2021-05-27

**Authors:** Madalina Ciortan, Matthieu Defrance

**Affiliations:** Interuniversity Institute of Bioinformatics in Brussels, Université Libre de Bruxelles, Brussels, Belgium

**Keywords:** Single cell, sc-RNA seq, Clustering, Contrastive learning, Self-supervised representation learning, Neural networks, Deep learning, Optimization

## Abstract

**Background:**

Single-cell RNA sequencing (scRNA-seq) has emerged has a main strategy to study transcriptional activity at the cellular level. Clustering analysis is routinely performed on scRNA-seq data to explore, recognize or discover underlying cell identities. The high dimensionality of scRNA-seq data and its significant sparsity accentuated by frequent dropout events, introducing false zero count observations, make the clustering analysis computationally challenging. Even though multiple scRNA-seq clustering techniques have been proposed, there is no consensus on the best performing approach. On a parallel research track, self-supervised contrastive learning recently achieved state-of-the-art results on images clustering and, subsequently, image classification.

**Results:**

We propose *contrastive-sc*, a new unsupervised learning method for scRNA-seq data that perform cell clustering. The method consists of two consecutive phases: first, an artificial neural network learns an embedding for each cell through a representation training phase. The embedding is then clustered in the second phase with a general clustering algorithm (i.e. KMeans or Leiden community detection). The proposed representation training phase is a new adaptation of the self-supervised contrastive learning framework, initially proposed for image processing, to scRNA-seq data. *contrastive-sc* has been compared with ten state-of-the-art techniques. A broad experimental study has been conducted on both simulated and real-world datasets, assessing multiple external and internal clustering performance metrics (i.e. ARI, NMI, Silhouette, Calinski scores). Our experimental analysis shows that *constastive-sc* compares favorably with state-of-the-art methods on both simulated and real-world datasets.

**Conclusion:**

On average, our method identifies well-defined clusters in close agreement with ground truth annotations. Our method is computationally efficient, being fast to train and having a limited memory footprint. *contrastive-sc* maintains good performance when only a fraction of input cells is provided and is robust to changes in hyperparameters or network architecture. The decoupling between the creation of the embedding and the clustering phase allows the flexibility to choose a suitable clustering algorithm (i.e. KMeans when the number of expected clusters is known, Leiden otherwise) or to integrate the embedding with other existing techniques.

**Supplementary Information:**

The online version contains supplementary material available at 10.1186/s12859-021-04210-8.

## Background

Single-cell RNA sequencing (scRNA-seq) provides transcriptional profiling of individual cells, enabling researchers to study the transcription dynamics, the composition of tissues or the relationships within gene-networks [1]. In the absence of cell type annotations, unsupervised clustering models are typically employed to identify or discover cellular subtypes in scRNA-seq data. Despite the extensive study of clustering models in machine learning [2, 3], single-cell transcriptomic clustering remains challenging due to the high dimensionality of data (the number of transcripts is usually greater than 20,000, leading to “the curse of dimensionality”), the high sparsity due to low mRNA expression level and dropout events.

During the last decade, numerous clustering methods emerged to propose diverse solutions to the technical challenges raised by scRNA-seq data analysis, as shown in review papers [4–7]. CIDR [8] started by addressing the dropout problem with a data imputation phase before clustering the PCA-reduced representation using hierarchical clustering. RaceID [9] has been customized for identifying rare cell types improving the clustering performance by replacing KMeans with K-medoids. SIMLR [10] learns a similarity measure between cells using multiple kernels and performs spectral clustering on this robust distance metric. ScRNA [11] applies transfer learning to unsupervised clustering problems by incorporating information from a larger annotated dataset via non-negative matrix factorization. SOUP [12] allows to cluster both pure and transitional cells by leveraging soft cluster memberships, computed based on the expression similarity matrix. Seurat [13] performs a cell-community detection on top of the shared nearest neighbor graph, using the Louvain algorithm. Several scRNA-seq analysis methods, including Seurat, have been made available in the python package scanpy [14].

More recently, deep learning techniques have been adapted to analyzing scRNA-seq data. A deep count autoencoder, DCA [15], was proposed to denoise and impute scRNA-seq data by learning three components: (1) the count distribution, (2) the sparsity and (3) the overdispersion. DCA proposed to approximate the zero-inflated negative binomial (ZINB) distribution of the expression data using an autoencoder model. The autoencoder consists of 3 output layers, each corresponding to one of the three components (1–3). This architecture became a baseline for several other state-of-the-art clustering methods. ScDeepCluster [16] enriched the DCA model with a clustering layer attached to the autoencoder’s learned embedding space (i.e. the bottleneck layer). This approach followed the DEC [17] method, initially proposed for image and text unsupervised analysis. ScDeepCluster employs as loss a linear combination of the ZINB loss and the Kullback–Leibler (KL) divergence between the distribution of soft labels of the embedding layer (measured by a Student’s t distribution) and the derivation of the target distribution. This combined loss helped to preserve the local structure of the data generating distribution while refining the clusters. The DESC model [18] followed a similar approach as scDeepCluster but separated the data construction from the clustering phase. ScziDesk [19] proposed a weighted soft KMeans clustering (instead of hard clustering) to enhance similar cells' association under the same cluster. ScziDesk employs as loss function a linear combination of (1) the ZINB loss, (2) a weighted soft KMeans loss and (3) a KL divergence between the Student’s t distribution of the embedding space and that of a target distribution, as proposed in DEC [17]. The weights in the soft KMeans were computed with a Gaussian kernel function, assessing each cell's proximity to the cluster center. The KL divergence loss was based on the pairwise similarity of data points in the latent space and encouraged similar points to be clustered to the same cluster. ScVI [20] is another neural-network approach to approximate the underlying ZINB distribution of the observed expression values; it performed several tasks such as batch correction, clustering, differential expression and visualizations.

Despite the abundance of clustering methods, there is no consensus regarding the best approach under every circumstance. Freytag et al. showed in the Cluster Headache publication [6] that 11 state-of-the-art methods for scRNA-seq clustering produced different results, also having little in common with a supervised labeling approach. This analysis highlights well the challenges of the field.

## Methods

In this work, we propose an unsupervised deep learning method to cluster scRNA-seq data using contrastive representation learning. Our method, *contrastive-sc*, analyzes the expression count matrix $D=\{{x}_{ij}\}\in {\mathbb{R}}^{n*d}$(having ***n*** samples [i.e. cells] and ***d*** features [i.e. transcripts]) in a two-phased process in order to identify clusters of well-separated groups of cells. In a nutshell, an artificial neural network (the encoder model) is trained to produce representations (embeddings) for each cell which is then clustered in a second phase with a general clustering algorithm. The training of the encoder model follows the contrastive representation learning framework which was detailed below.

### Data preprocessing

Our method adopts the preprocessing phase proposed in scziDesk [19] and implemented in the python package scanpy [14]. First, the genes expressed in only one cell or less are discarded. Next, the expression count matrix is normalized by the library size so that the total counts are identical across cells. The scanpy library implements this by dividing each cell by the sum of all its count values and then multiplying it by the median of all cells' total expression values. A natural logarithm is applied to the normalized data. Next, as proposed in scziDesk, only the most variable genes (i.e. top 500 genes) are selected according to their dispersion ranking, computed by scanpy following [13]. This selection step maximizes the underlying information in the retained genes while significantly reducing the computational load. Finally, the data are scaled such that each gene has zero mean and unit variance. The result of the preprocessing phase was used as input to the predictive model, described below.

### Representation learning

**Fig. 1:**
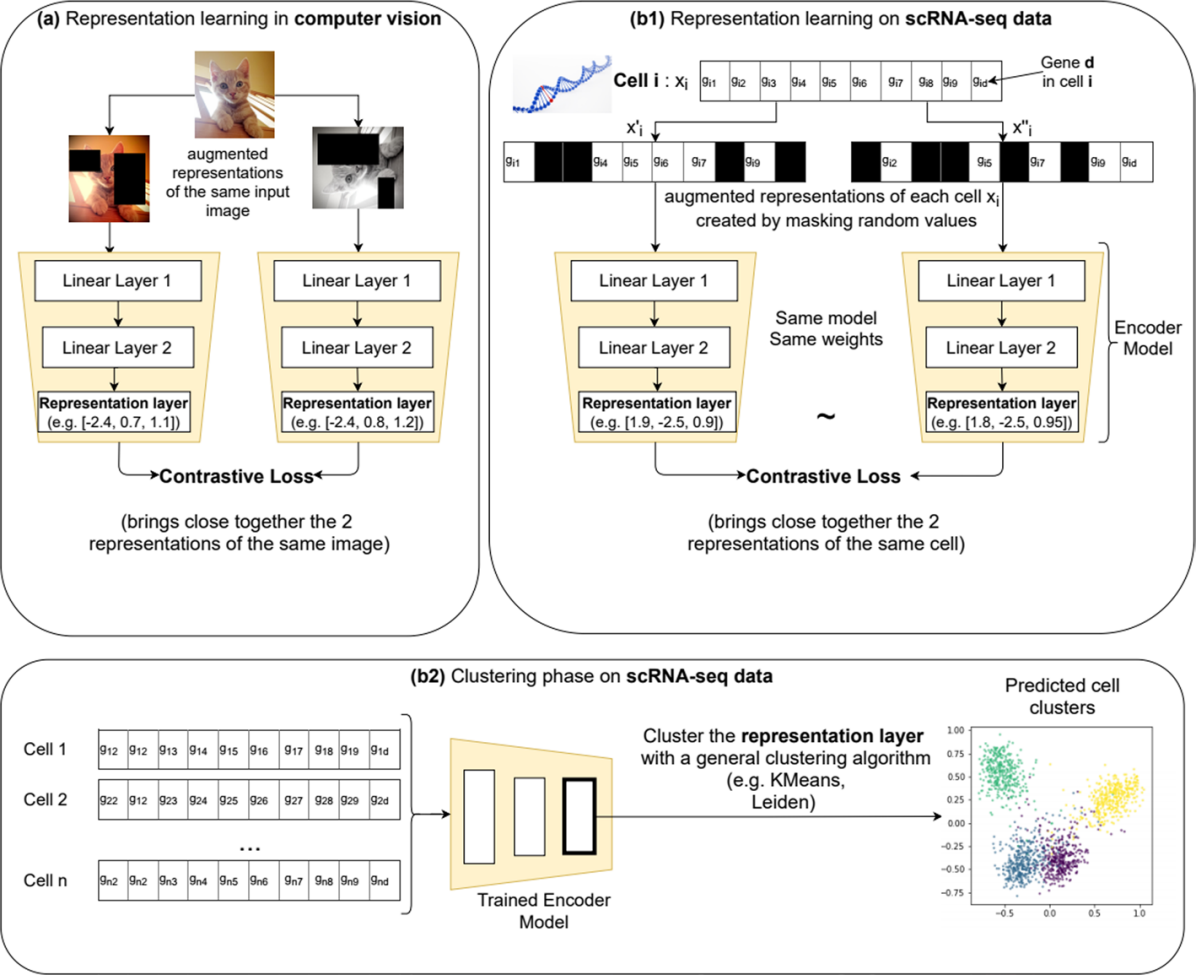
Method overview. The method is inspired by the contrastive learning techniques proposed for image analysis (**a**). For each image, an embedding (i.e. the value of the representation layer) is learned by applying a contrastive loss on the representations from 2 copies of the same image, modified with strong transformations such as multiple cropping, pixel noise, rotations, translations. This embedding can be analyzed with a general clustering algorithm in order to produce cluster assignments for each image. A similar process was proposed for scRNA-seq data (**b1**, **b2**): first, a representation learning phase (**b1**), produces an embedding for each cell (e.g. the vector [2.3, − 3.1, 0.2] is the embedding for the depicted Cell i). After training the network, all generated cell embeddings are clustered with a general clustering algorithm like KMeans or Leiden (**b2**). The representation learning starts from two strongly augmented copies of the input data ($x^{\prime}_i$ and $x^{\prime\prime}_i$) created by masking an arbitrary number (e.g. 80%) of input genes, denoted as [g1, …, gd]. The network is trained with an unsupervised contrastive loss, guiding the model to map the similar views to neighboring representations and the dissimilar to non-neighboring representations

Self-supervised contrastive learning is a representation learning technique that has been recently explored in the context of computer vision, where it typically produces representations (embeddings) for unlabeled images. The resulting embeddings can be either clustered directly or, if a set of labels is available, a classification layer can be added and trained accordingly. Self-supervised contrastive learning produced state-of-the-art results in semi-supervised [21] and unsupervised (clustering) [21, 22] settings. The training process consists of creating augmented versions of each image, which, in combination with the contrastive loss [23] (detailed in Additional file 1), pushes closer together (in the representation space) the augmented versions of the same image and farther away from all other images. Several contributions [21, 23, 24] reported that stronger image augmentations bring a significant performance gain. In image analysis, traditional image augmentations represent transformations such as rotations, translations or blurring. Strong data augmentations change the original image significantly, for instance, by cropping multiple random portions or applying high levels of noise, as illustrated in Fig. 1a.

In this work, we explored the application of self-supervised contrastive learning, typically employed for image processing, to scRNA-seq data. As illustrated in Fig. 1b1, for each input cell ${x}_{i}$ two distinct augmented *views* of the same data are created (i.e. the pair of samples from the same class : $x_{i}^{\prime}$ and $x_{ii}^{\prime \prime}$). As many of the transformations available for images (i.e. change in colors, rotations, translations) do not have an equivalent on scRNA-seq data, our augmentation technique consists primarily in masking an arbitrary random set of genes in each view so that they are ignored from the underlying computation. This technique was implemented by using a dropout neural network layer [25] directly on the input data, which assigned a weight of 0 to randomly selected gene values. For disambiguation, the name of the neural-network layer (dropout) is not related to the false zero counts affecting scRNA-seq data (also named dropout); the former will be referred to as neural network dropout or NN dropout. Other data augmentation techniques such as adding random Gaussian noise to each view have been explored and presented in the results section but did not provide a performance gain.

Representation learning was performed with a single artificial neural network, constituting the encoder model. Each of the two augmented copies of input cells was processed by the same encoder model trained to minimize the contrastive loss proposed in [23] (see details in Additional file 1) and, thus, the distance between the augmented copies. At each iteration, different randomly augmented versions were generated for each cell, which exposed the model to a wide variety of augmentations and prevented the memorization of a particular instance. The output of the last hidden layer of the encoder, also named the representation layer, provides an embedding (a vector representation) for each cell. The architecture of the encoder model consists of several stacked linear layers. An extensive neural architecture search presented in the results section identified the optimal architecture consisting of 3 layers of size [200, 40, 60], thus producing a cell embedding of 60 values.

Unlike images, scRNA-seq data is affected by dropout events, representing missing gene measurements that produce incorrect zero count observations. Existing scRNA-seq clustering methods start by addressing the dropout using models such as NB or ZINB autoencoder [15, 19, 26], which models the expected negative binomial or zero inflated negative binomial distribution of counts data. Our method pursues an alternative approach to this analytical framework and relies primarily on the strong data augmentation to acquire robustness to dropout without performing an explicit imputation before the clustering step.

### Clustering phase

After the representation learning phase produced cell embeddings, a general clustering algorithm (i.e. KMeans, Leiden [27]) can be employed to obtain cell-cluster assignments. The decoupling between the embedding creation and the cluster assignment provides flexibility to adapt to both cases when the expected number of clusters is known (as required by KMeans) or unknown (Leiden community detection), as well as incorporating any other suitable clustering technique.

## Results

Clustering analysis is typically performed when no class membership annotations (i.e. the ground truth labels) are available. From the point of view of the end-user, two scenarios can be distinguished: (1) an exploitation setting, in which searches start from a prior knowledge or a good definition of the sample groups to be identified and (2) an exploratory setting when no prior expectations about the number or the size of the data clusters exist. This distinction has been made because some of the existing libraries require to input the number of clusters to be identified while others can dynamically infer it from various data density or connectivity criteria. However, the first category of libraries can still be used in an exploratory setting by computing the clustering several times (each for a number of clusters in a plausible range of values) and selecting the best result. The selection typically maximizes internal quality measures, assessing if the identified clusters are compact and well defined. However, this parameter exploration process introduces an additional cost in terms of computation time, complexity, and, as detailed below, there is no consensus on the best internal quality score to employ.

### Evaluation scores

The evaluation of clustering performance has been made using four metrics: Adjusted Rand Index (ARI) score [28], Normalized Mutual Information (NMI), Silhouette score [29] and Calinski and Harabasz [30]. For all selected scores, the higher the value, the better the performance. This detailed strategy has been implemented because, contrary to supervised analysis, there is no consensus on the optimal measure to evaluate clustering methods [31]. Secondly, in a typical scenario, ground truth information about the cluster assignment is not available, and as such, requires to optimize various internal quality scores. For example, Silhouette scores measure the predicted clusters' compactness and produce values between − 1 and 1; the higher the score, the denser and better separated the clusters are. Calinski Harabasz score represents the ratio of the sum between cluster dispersion and within-cluster dispersion for all clusters and produces positives scores that are not bounded. However, various clustering methods may create different data projections, each with well-defined clusters, and still, the resulting partitioning can be significantly different from one another, especially when working with scRNA-seq data [6]. To mitigate this relativism, the main alternative is to employ for validation purposes datasets having ground truth annotations, which allows to compute external measures such as ARI or NMI. The former produces scores from − 1 to 1 and is proportional to the number of sample pairs whose labels are the same in the annotation and the model prediction, while NMI measures the agreement of the true and predicted cluster assignments, ignoring permutations. Moreover, most clustering publications [16, 19] report their results primarily in terms of one or several external quality measures, while the internal quality analysis is reduced to a visual comparison of the 2D representation of identified clusters. To keep the presentation of results easy to follow, for some experiments only the ARI and/or Silhouette scores have been included in the main manuscript. The complete assessment is provided in Additional file 1.

### Competing methods

An extensive empirical study has been carried out by comparing the performance of *contrastive-sc* with 11 alternative techniques, representing both methods requiring or not the number of clusters as input. ScziDesk [19], scDeepClustering [16], scRNA [11], cidr [8] and soup [12] take as input the expected number of clusters while Seurat [13] (scanpy [14] implementation), desc [18], scedar [32], raceid [33] and scvi [20] perform clustering without any alternative information. Additionally, a naive baseline method consisting of clustering with KMeans the first 2 principal components of the expression matrix has been assessed. A detailed record of all benchmarked methods and their repositories has been made available in Additional file 1: Table S1. The testing of all methods has been performed by employing their default parameters proposed in the issuing paper or in the official repository listed in Additional file 1: Table S1. The code used for executing each method has been made available on GitHub, along with the underlying results. As highlighted in Additional file 1: Table S1, half of the methods are available in python and the other half in R. To facilitate the reproducibility of the analysis, two docker containers have been made available for each environment. For statistical significance, all experiments have been performed three times, and the reported result represents the average score. To provide a rapid assessment of each method’s stability, most results have been presented as bar plots, highlighting the variation of results across runs.

### Implementation

*contrastive-sc* has been implemented in Python 3 using the deep-learning framework pytorch [34]. The clustering algorithms use publicly available implementations for KMeans^1^ and Leiden^2^ community detection. The Encoder network consisted of 3 linear layers of [200, 40, 60] neurons and produced cell embeddings in 60D. The neural network was trained with the Adam [35] optimizer using an initial learning rate of 0.4 and a default cosine scheduler. The model training was carried out for 30 epochs using randomly sampled mini-batches with 200 cells. Similar to scziDesk, our method employed a preprocessed dataset with the 500 most variable genes for simulated and biological datasets. All our experiments continued the representation phase by clustering the embedding with both KMeans (labeled Contrastive + KM) and Leiden community detection (Contrastive + LD). Our tests were executed on 1GPU GeForce RTX 2060. For a complete computational assessment, our method was equally benchmarked on CPU. The experimental setting consisted of analyzing a collection of 24 simulated and 15 real scRNA-seq datasets, as detailed below.

### Analysis of simulated data

The data simulation strategy consisted of generating balanced and imbalanced datasets (i.e. uniform and non-uniform distribution of cluster sizes). The R package splatter [36] has been used to produce datasets approximating various biological scenarios in which we controlled the number of clusters, samples, genes and dropout rates. The same experimental setting (i.e. the same parameters for splatter) as scziDesk and scDeepCluster has been reused to facilitate comparisons, but we extended the range of explored parameters to assess the methods' behavior under new conditions. The number of clusters per dataset was extended from 7 to 16, to evaluate the impact of a growing number of clusters on the method performances. The dropout rate was extended from 30% to approximately 40% to study the model performances under more severe dropout conditions.

The balanced datasets consist of 2500 genes, 4, 8 and 16 clusters and dropout rates ranging from 5 to 38% (splatter parameters being shape =  −1, type = “experiment”, facScale = 0.2 and mid in [−1, 0, 1, 1.5]). Note that the dropout rates are estimated by the library for each input *mid* parameter and are inferior to the data sparsity (the number of 0 values in the expression matrix), as scRNA seq data also contains a significant number of 0 values associated with the genes not expressed in the studied cells. A constant cluster size of 250 samples has been employed on balanced datasets, and the total sample size is thus proportional to the number of embedded clusters (i.e. 4 × 250 to 32 × 250). The imbalanced datasets consist of 2500 genes, 3000 cells, 4, 8 and 16 clusters with size ratios from 0.6 to 0.01 and dropout rates from 5 to 38%.

**Fig. 2:**
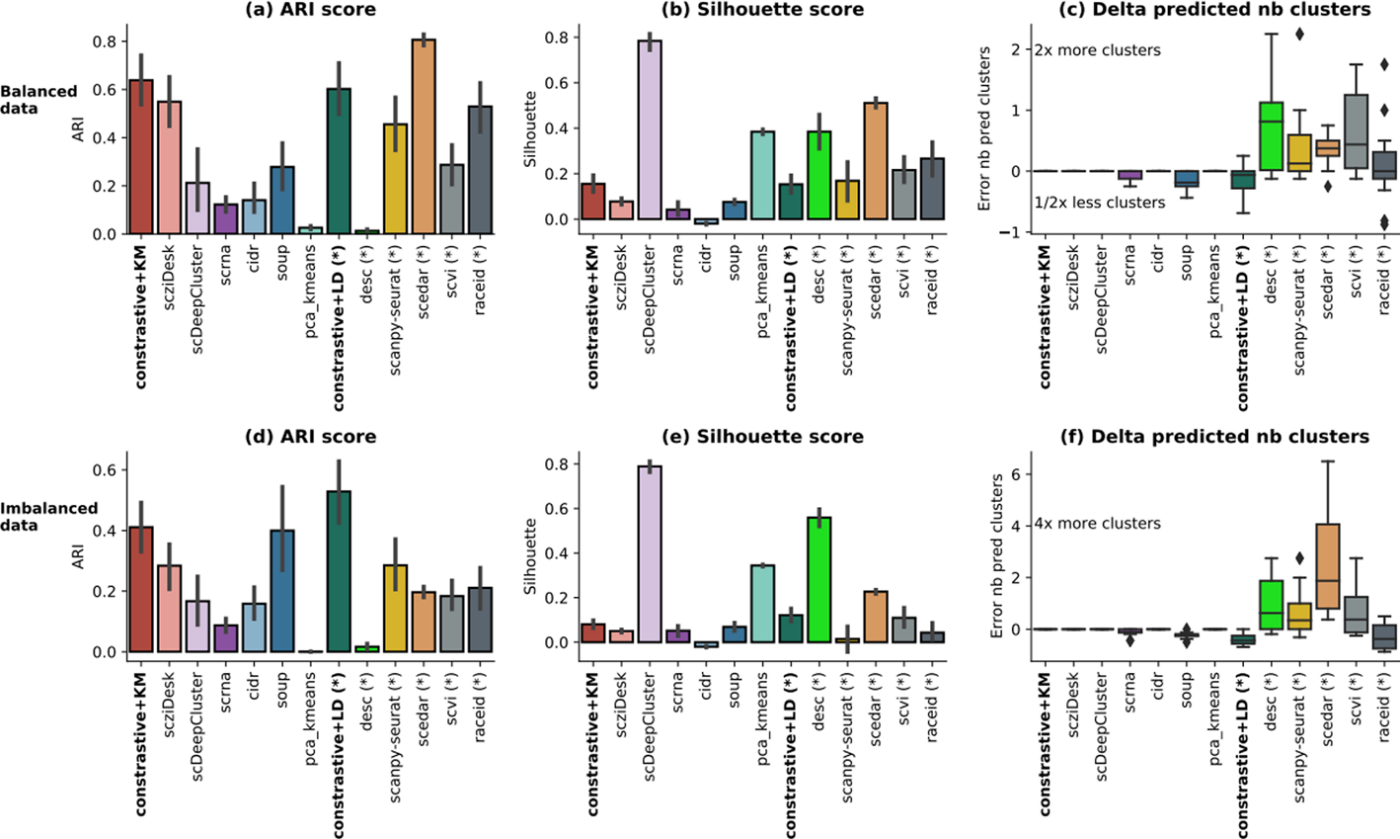
Simulated data analysis. A set of 12 simulated balanced (**a**–**c**) and 12 imbalanced (**d**–**f**) datasets has been analyzed. For simplicity, only one external (ARI) and one internal (Silhouette) evaluator across all datasets are displayed. The complete analysis is provided in Additional file 1: Fig. S1, S2. Each method processed each dataset 3 times with different initialization seeds. The error as the relative difference between the predicted and the true number of clusters [(pred − true)/true] is illustrated in **c** for balanced data and **f** for imbalanced data. The methods annotated with (*) are those that did not receive as input the number of clusters. Most methods in this category tend to overestimate the number of clusters in the data, behavior which is more pronounced in the imbalanced setting

The scores on balanced data (Fig. 2a) are generally higher than those on imbalanced data (Fig. 2d), indicating that the latter raises a technical challenge to most techniques. The methods that did not use the number of clusters as input parameter, (annotated with * in the figure) overestimate it up to 2 times the actual value (i.e. 4, 8 and 16 clusters) on balanced data and more significantly on imbalanced data (Fig. 2f). In turn, this behaviour penalizes the external score and explains the relative loss of performance in the imbalanced setting. On the other hand, these methods (*) provide higher internal scores on Silhouette (Fig. 2b, e) and Calinski metrics (Additional file 1: Fig. S2d, S2h). *contrastive-sc* provides encouraging results in both balanced and imbalanced settings. The two clustering implementations (KMeans and Leiden) display similar results on balanced data, but in the imbalanced setting, the Leiden community detection provides on average the best results across all competitors. In both settings, contrastive + Leiden tends to under-estimate the number of clusters. However, this behavior can be fine-tuned by changing the input neighborhood size parameter in the Leiden clustering execution. A dataset-level analysis has been performed (Additional file 1: Fig. S1) and confirms the speculation that most methods suffer significant degradation in performance when the dropout rate or the number of clusters increases. The method ranking per dataset (Additional file 1: Fig. S1) indicates there is no constant best method across all experiments, however scedar, scanpy-seurat, soup, scziDesk, and our methods provided repeatedly the top results.

As simulated datasets remain an approximation of the biological data, the following part of the article focuses on the analysis of a collection of 15 real-world datasets.

### Analysis of scRNA-seq datasets

The scRNA-seq datasets made available in scziDesk and scDeepCluster have been combined to produce a collection of 15 real-world datasets to benchmark the model performance. The datasets from scziDesk have been assembled at Stanford University from mouse scRNA-seq data for various organs using both Smart-seq2 and 10 × Genomics sequencing [37]. The Smart-seq2 datasets have been prefixed with “Quake Smart” while the latter with “Quake10 ×”. Other publicly available datasets have been added, as follows: Adam [38], Muraro [33], Romanov [39] and Young [40]. The scDeepCluster data had been collected using four sequencing platforms: 10 × genomics platform for the PBMC cells [41], droplet barcoding for mouse embryonic stem cells [42], Microwell-seq for mouse bladder cells [43] and sci-RNA-seq for worm neuron cells [44]. As detailed in Table 1, all datasets are imbalanced and contain 4–16 annotated clusters across 870 to 9552 cells. Further details about data sparsity and other descriptive statistics specific to each dataset can be found in Additional file 1: Table S4.

**Table 1:** Description of scRNA-seq datasets

	Dataset name	Size (cells × genes)	Number of clusters	Cluster sizes
1	Quake Smart seq2 Trachea	1350 × 23,341	4	(830, 206, 201, 113)
2	Quake10 × Bladder	2500 × 23,341	4	(1203, 1167, 73, 57)
3	Quake10 × Spleen	9552 × 23,341	5	(6886, 1930, 464, 230, 42)
4	Quake Smart seq2 Diaphragm	870 × 23,341	5	(439, 241, 81, 78, 31)
5	Quake10 × Limb Muscle	3909 × 23,341	6	(1330, 1136, 461, 354, 320, 308)
6	Quake Smart seq2 Limb Muscle	1090 × 23,341	6	(540, 258, 141, 71, 45, 35)
7	Romanov	2881 × 21,143	7	(1001, 898, 356, 267, 240, 71, 48)
8	Adam	3660 × 23,797	8	(629, 617, 516, 513, 463, 396, 302, 224)
9	Muraro	2122 × 19,046	9	(812, 448, 245, 219, 193, 101, 80, 21, 3)
10	Young	5685 × 33,658	11	(1498, 1201, 731, 621, 483, 373, 268, 259, 118, 73, 60)
11	Quake Smart seq2 Lung	1676 × 23,341	11	(693, 423, 113, 90, 85, 65, 57, 53, 37, 35, 25)
12	10 PBMC	4271 × 16,653	8	(1292, 702, 606, 459, 450, 332, 295, 135)
13	Mouse ES cells	2717 × 24,175	4	(933, 798, 683, 303)
14	Worm neuron cell	4186 × 13,488	10	(1015, 842, 443, 400, 334, 314, 305, 239, 224, 70)
15	Mouse bladder cell	2746 × 20,670	16	(717, 357, 344, 316, 236, 224, 131, 80, 75, 64, 44, 41, 38, 36, 30, 13)

The datasets made available in scziDesk have been combined with those in scDeepClusters to create a wider benchmark of 15 datasets

**Fig. 3:**
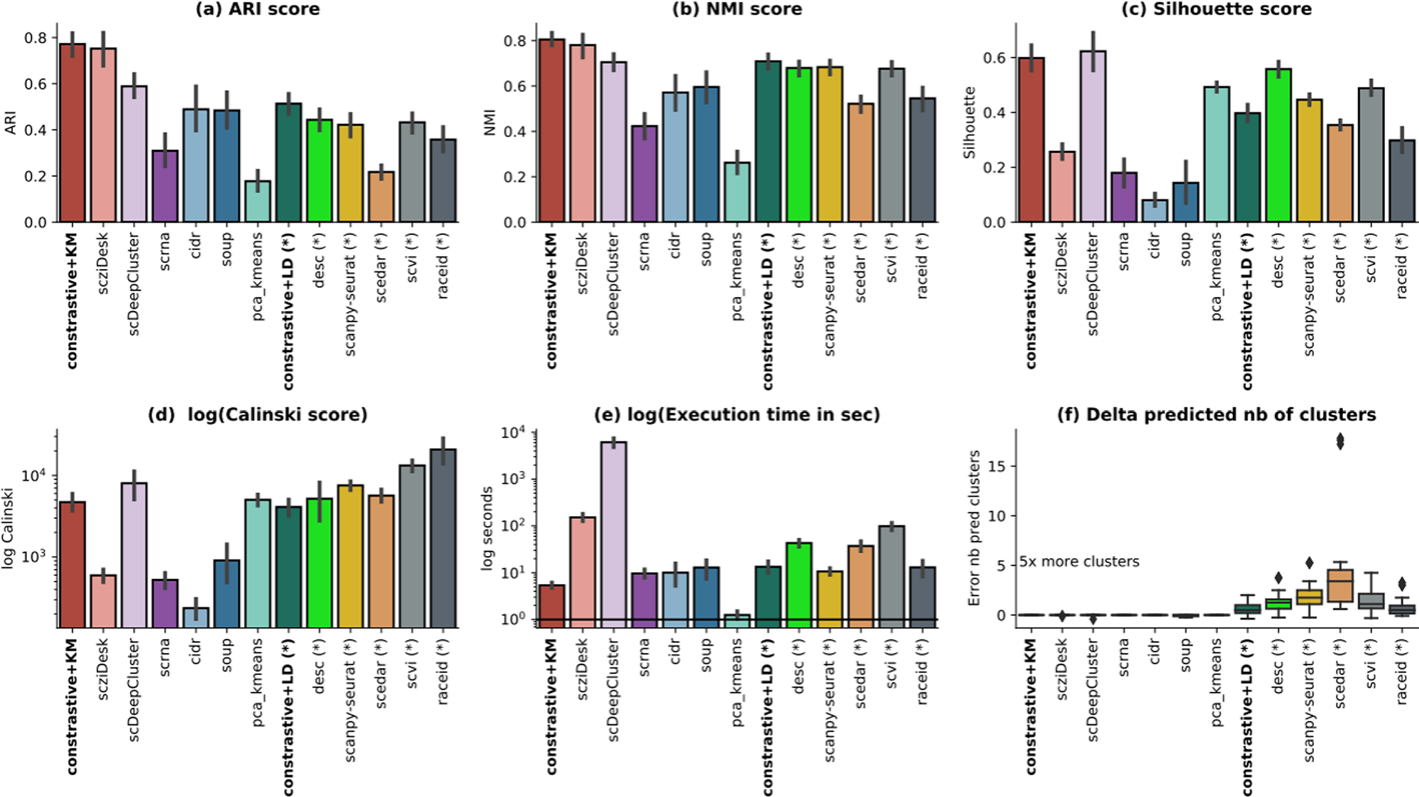
Real scRNA-seq data analysis. A method comparison has been performed across all 15 real datasets in terms of ARI—Adjusted Rand Index (**a**), NMI—Normalized Mutual Information (**b**), Silhouette (**c**), Calinski (**d**) scores, the execution time (**e**) and the error of the predicted number of clusters (**f**). The execution time and the Calinski scores have been depicted in log scale. The error as the relative difference between the predicted and the true number of clusters [(pred − true)/true)] is illustrated in **f**. The methods annotated with (*) indicate those where the clustering was performed without inputting the number of clusters. All methods in this category tend to overestimate the number of clusters in the data and do more so in the imbalanced setting. For each dataset and method, three runs have been performed

The average results across all methods have been summarized in Fig. 3, depicting the 4 clustering measures, the execution time and the error in the estimated number of clusters. Our methods compared favorably to state-of-the-art techniques (scziDesk, scDeepCluster, scanpy-seurat, scedar) across all clustering scores. Clustering the learned embedding with KMeans performs better than the Leiden community clustering. The latter overestimates the number of identified clusters by 1.65 times (Fig. 3f), penalizing the external scores. On average, the partitions identified with *constastive* + *KMeans* agree most with ground truth (average ARI is 0.77 and NMI 0.81), but they also have a good internal quality as indicated by the Silhouette (0.6) and Calinski scores (4100). Moreover, in terms of execution time, *constastive-sc* + *KMeans* is the fastest method from all state-of-the-art techniques with an average execution time of 5.29 s, surpassed only by the naive baseline. A detailed computational analysis is depicted in the next section.

As expected, the worst-performing method is the naive baseline, consisting of clustering with KMeans, the first two principal components of the expression matrix. The best performing methods on the benchmarked real-world datasets use the number of clusters to be identified while running KMeans related heuristics: scziDesk, scDeepCluster. The other methods (annotated with *) have a significant tendency to overestimate the number of clusters in the data, on average by a factor of 2 (desc, scanpy-seurat, scvi, raceid), but up to 5 times (scedar); however, the identified partitions have generally higher internal quality scores, as indicated by both the Silhouette (Fig. 3c) and Calinski (Fig. 3d) scores. In some cases, this behavior may be attenuated with an additional work of method-specific hyper-parameter tuning for each dataset, but this introduces additional computational load and requires defining an experimental setup adapted for each technique, which goes beyond the scope of a broad benchmarking exercise. For comparison, both *constrative-sc* methods used the same model hyper-parameters across all experiments. The most computationally expensive methods are scDeepCluster and scziDesk. Being based on neural networks, they perform a long pretraining phase (600 and 1000 epochs) before a final clustering fine-tuning. Unlike scziDesk, which employs the top 500 most variable genes and thus reduces the input data's dimensionality, scDeepCluster employs all genes, thus explaining the associated peak in computational time. The external quality measures (ARI and NMI) are aligned across all methods, as indicated in Fig. 3a, b and confirmed computationally by significant correlation values (above 0.86 Pearson coefficients as per Additional file 1: Fig. S5a). However, as depicted in Fig. 3c, for some methods (desc, scDeepCluster), the best-separated cell partitioning is not always aligned with the ground truth annotation, and conversely. This observation is confirmed computationally by the diverse correlation levels between external and internal scores, computed per method (from − 0.26 for desc to 0.9 for raceid Additional file 1: Fig. S5a).

**Fig. 4:**
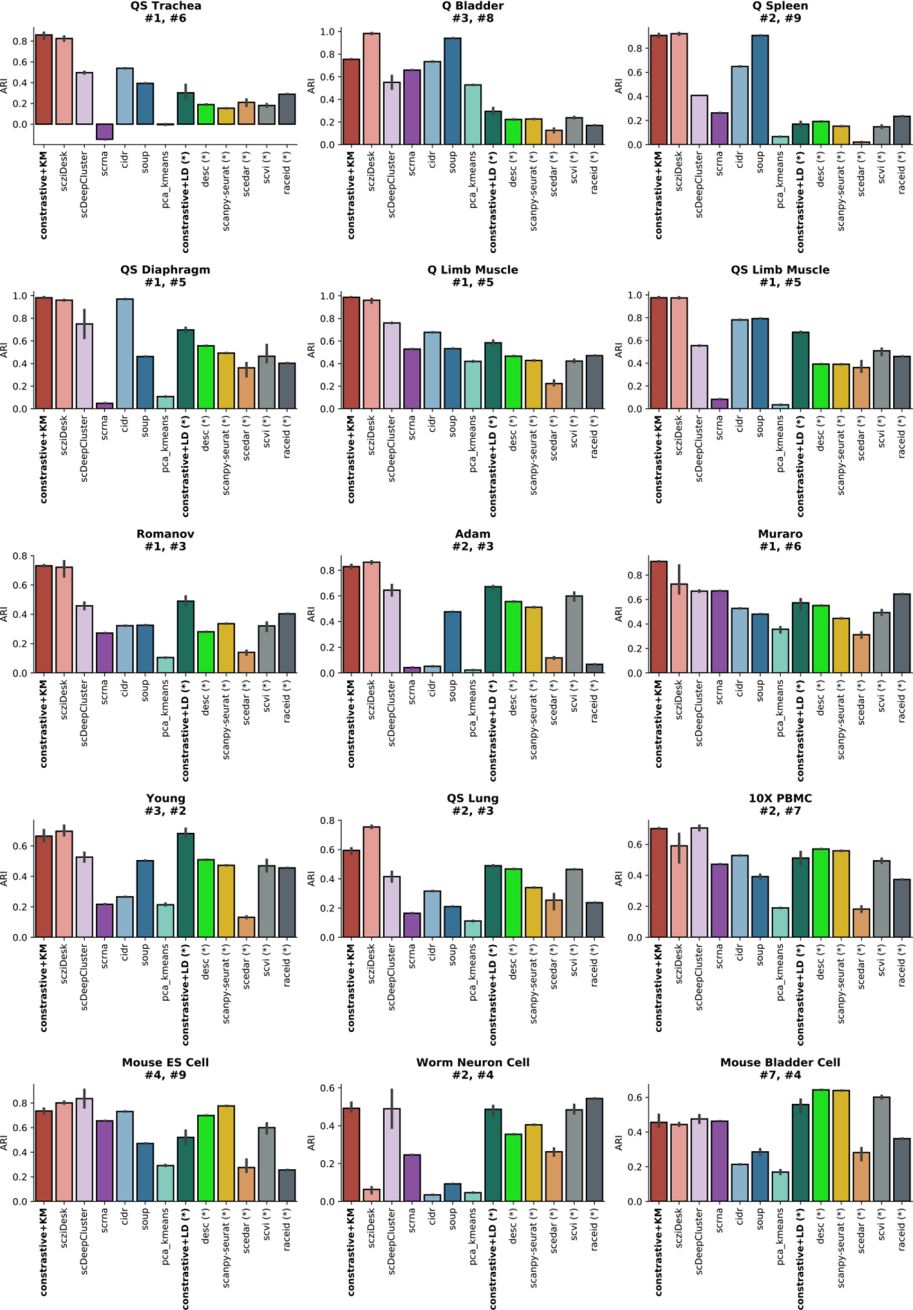
Dataset-level analysis of real scRNA-seq data on ARI (Adjusted Rand Index) scores. The results aggregate 3 consecutive runs of all 13 explored methods over the 15 biological datasets. The dataset annotations (e.g. #1, #6) indicate the ranking of contrative-sc with KMeans and Leiden on each analyzed dataset: the best performing method 6 datasets and the second best on another 6

Next, a dataset-level method comparison on real-world datasets has been performed on ARI scores (Fig. 4). The results indicate no consensus regarding the best method, the performance depending on the specificities of the analyzed dataset. *contrastive-sc* provides better results when using KMeans than when using a default configuration for Leiden community detection, which generally identifies smaller sized clusters. Further dataset level hyperparameter tuning for Leiden will be explored in future works. However, our method compares favorably with the best performing techniques (scziDesk, scDeepCluster, desc, scanpy-seurat): it provided the highest score on 6 datasets, the second-best on another 5 from a total of 13 explored techniques. A detailed analysis of the underlying internal quality of identified clusters has been performed (Additional file 1: Fig. S3). For simplicity, the comparison focuses only on the best performing methods (scziDesk, scDeepCluster, desc, scanpy-seurat) on the benchmarked datasets. Both Silhouette and Calinski scores indicate that clusters identified by *contrastive-sc* are generally well defined. Desc and scDeepClusters identify the best-separated data partitions; however, they are not always in agreement with the ground truth.

### Selection of input genes

**Fig. 5:**
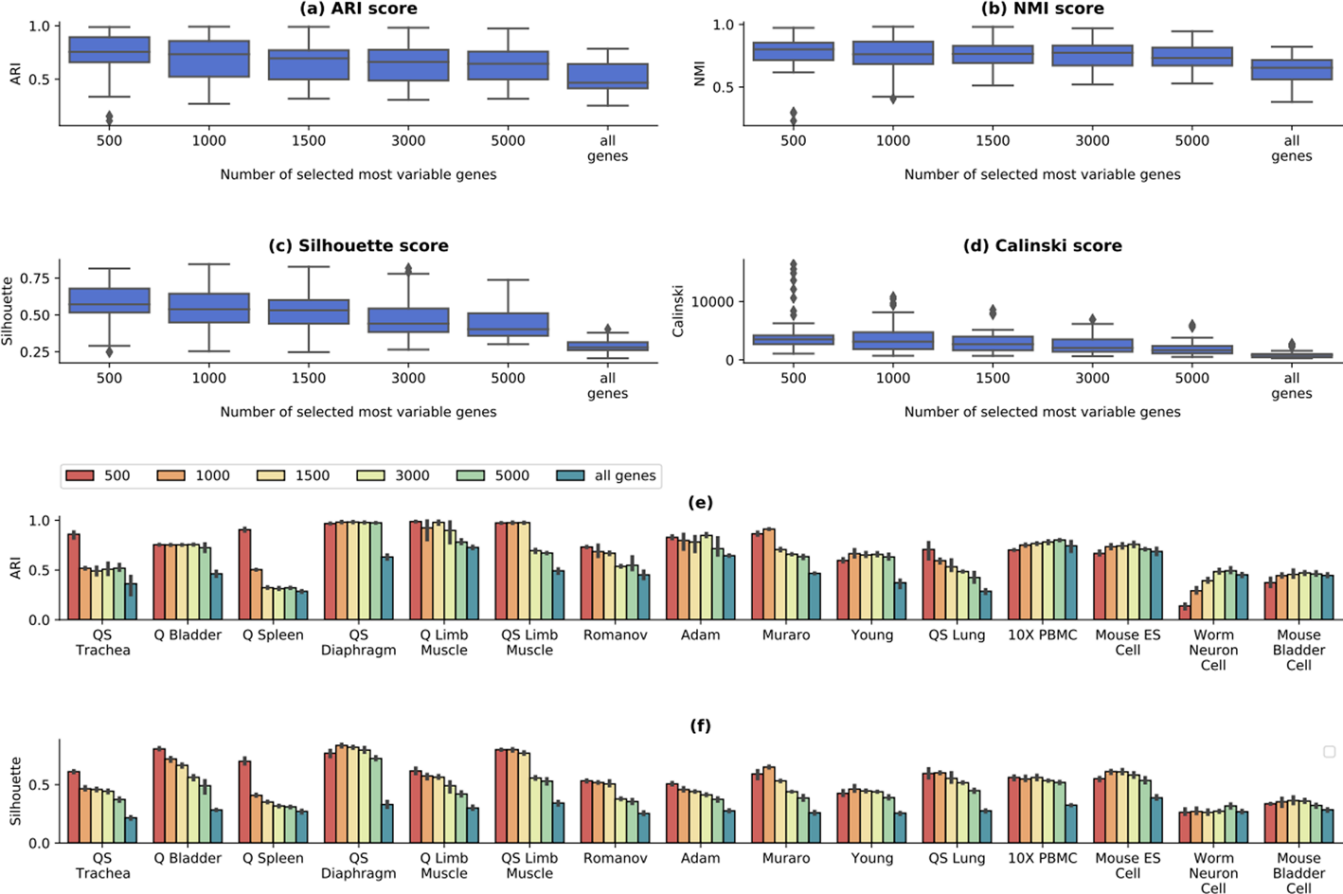
Gene selection analysis on real data. The selection of the top variable genes (500–5000) was compared with no selection (all genes). The plots depict 3 runs on each of the 15 real datasets on all computed scores (**a**–**d**). On average, best scores are achieved using the top 500 genes. Both the internal and external quality decline when using more than 1000 genes, which corresponds to including many genes with low levels of expression. The dataset-level results, depicted as ARI (**e**) and Silhouette scores (**f**), indicate that for some datasets, a significant gain in performance can be attained when using up to 5000 genes (e.g. Worm Neuron Cell dataset)

A comprehensive study on the importance of gene selection has been performed in scziDesk, comparing the performance obtained by selecting the top 500 most variable genes with that of scDeepCluster, where no such selection was performed. Their analysis reported a statistically significant gain between the original scDeepCluster and a version modified to select the top 500 most variable genes. In this section, a similar study has been performed on *contrastive-sc* by comparing the results of selecting most variable 500, 1000, 1500, 3000 and 5000 genes with selecting all genes (Fig. 5). The best performance is achieved when using the top 500 most variable genes, as suggested in scziDesk. Using the entire dataset brings a loss of performance which can be explained by the inclusion of low-expressed genes, more affected by dropout events. To assess the statistical significance of the performance gain brought by using the top 500 most variable genes instead of the entire dataset, a one-sided greater pairwise t-test was performed on the ARI and NMI scores of the 2 groups. The statistical tests reported *P* values of $5.15\times {10}^{-8}$ and $2.22\times {10}^{-5}$, thus confirming the importance of the gene selection step in the preprocessing phase.

To validate that selecting most variable 500 genes is the optimal setting for all datasets, a dataset-level visualization of the underlying ARI and Silhouette scores has been performed (Fig. 5a, c). For some datasets (e.g. Worm neuron cell, Mouse bladder cell) selecting the top 5000 genes provides a significant performance improvement both for the external and internal evaluators. For this reason, we took a step further from the default setting proposed in scziDesk and selected the dataset input size, which maximized the internal Silhouette score (Fig. 5c). For example, the Worm Neuron Cell dataset's optimal size is 5000 genes, while for Quake Spleen, it is 500. This improvement can be applied to any dataset, as it does not require ground truth annotations. The optimal parameters identified for each real-world dataset is provided in Additional file 1: Table S5. Input size optimization brings a performance boost to achieve an average of 0.77 ARI scores across all real datasets instead of a 0.75 ARI score when using the default value of 500 genes.

### Computational performance analysis

**Fig. 6:**
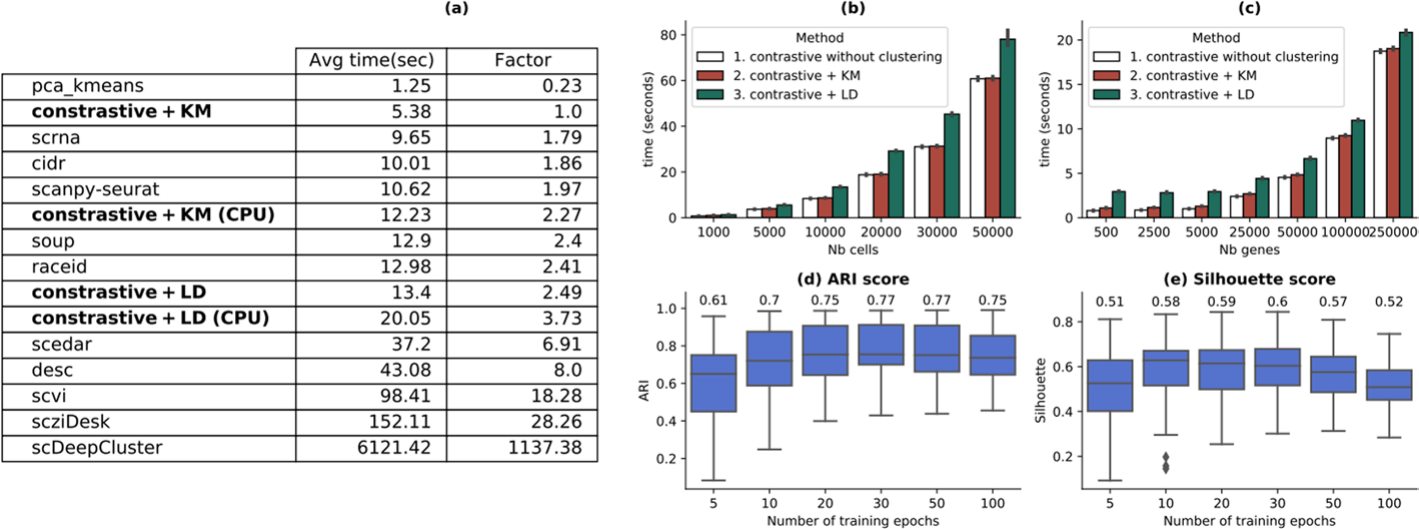
Execution time and scalability analysis. Average execution time for all benchmarked methods (**a**). The relation between the performance of contrastive + KMeans and the other methods has also been computed as a speed factor. All methods have been run on GPU. For comparison, the contrastive methods have also been benchmarked on CPU (contrastive + KM CPU and contrastive + LD CPU). **b** Depicts how our method scales with an increasing number of cells (from 1000 to 50,000); **c** illustrates how our method scales with an increasing number of input variables (from 500 to 250,000); the number of epochs needed for *contrastive-sc* to reach maximum performance measured as ARI (**d**) and Silhouette (**e**) scores. The annotated values in **c** and **d** represent the mean score. For most datasets, 30 epochs are enough for the model to learn meaningful representations, which brings a computational speed gain compared to other deep-learning competitor methods. This plot depicts three runs of the proposed method for each of the selected numbers of epochs

This section compares the computational cost of *contastive-sc* with the other techniques and analyzes its scalability with increasing input size. The average run-time of all benchmarked methods on the real-world datasets (over 3 runs) has been summarized in Fig. 6a. From the selected state-of-the-art techniques, *contrastive* + *KMeans* has the highest computational efficiency, requiring, on average, 5.3 s to run on GPU and 12 s on CPU. Similar architectural methods (sczi, scDeepCluster, desc) require from 43 s to several orders of magnitude more execution time. The computation speed gain is explained by the short convergence time (30 epochs) in combination with a reduced number of selected input genes.

The scalability of *contrastive-sc* when increasing the number of input cells (from 1000 to 50,000) and input variables (from 500 to 250,000) is depicted in Fig. 6b, c. The maximum values in both these scenarios produced execution times on average less than 60 s per run. Additionally, Leiden community detection impacts the method's overall performance, which grows with the number of input cells (Fig. 6b). The number of features analyzed by both KMeans and Leiden is constant, as it represents the size of the representation layer (60 values). Thus, the variation of the number of input genes impacts only the representation learning phase's performance. The training time to learn meaningful representations has been analyzed (Fig. 6b, c) in terms of model performances recorded after an arbitrary number of epochs (ranging from 5 to 100), averaged over 3 different runs. Our results indicate that 30 epochs are enough to learn meaningful representations having good external and internal quality scores. Moreover, continuing the training beyond 30 epochs changed the external performance only marginally. The computational complexity of contrastive-sc consists of the computational complexity of training the neural network combined with the complexity of the clustering algorithm. The complexity of training the neural network grows linearly with the number of samples (cells) and can be estimated as *O*(*n* × *numberOfEpochs* × (*d* × *sizeLayer*1 + *sizeLayer*1 × *sizeLayer*2 + *sizeLayer*2 × *sizeLayer*3)), equivalent to $O\left(n\times 30\times \left(500\times 200+200\times 40+40\times 60\right)\right)$. As the structure of the network is constant, the computational complexity depends only on the number of input samples. If KMeans runs for t iterations, its computational complexity is $O(t\times k\times n\times d)$ where $k$ is the number of clusters.

The memory footprint of *contrastive-sc* consists of only 111.180 trainable parameters, while other deep-learning techniques such as scziDesk or scDeepCluster require 551.164 and, respectively, above 10.000.000 parameters (the latter grows with the number of genes).

### Biological interpretation of results

**Fig. 7:**
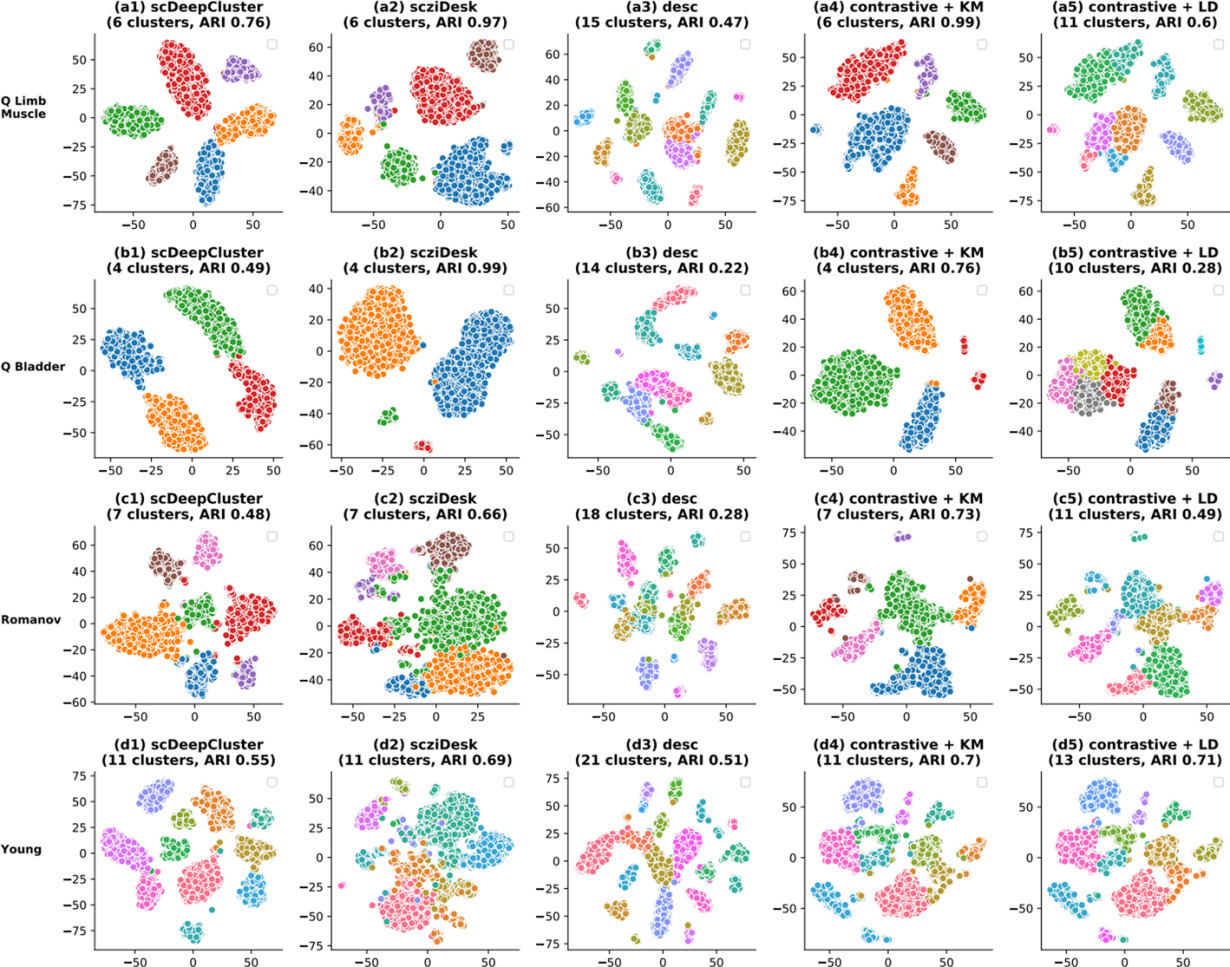
Visualization of identified clusters. scDeepCluster, scziDesk, desc, contrastive + KM, contrastive + LD identified clusters on 4 datasets (Quake Limb Muscle, Muraro, Romanov, Young) are compared using a 2D data projection. All selected methods start by creating an embedding for the cells which is clustered in a second phase. The quality of the method depends on both the created embedding and the clustering algorithm. For scDeepCluster and scziDesk, we depicted the clustering prediction relative to the underlying embedding. Our methods (contrastive + KM and contrastive + LD) clustered the same embedding. All plots present a 2D t-SNE projection of the underlying embeddings

To attach a biological interpretation to the cell clustering predicted by *contrastive-sc*, a set of 4 real-world datasets has been selected for visualization (Fig. 7). *contrastive-sc* has been compared, for simplicity, only with the best competitor methods: scziDesk, scDeepCluster and desc. The same embedding predicted by *contrastive-sc* has been clustered with KMeans and Leiden. Leiden tends to identify many small-sized clusters, and in doing so, it splits larger groups, thus penalizing the external score across most datasets. One exception is the Young dataset, containing a larger number of clusters (11). Leiden identified 13 clusters and achieved the highest performance from all explored methods (0.71 ARI). A similar behavior characterized the desc method, which overestimated up to 2 times the annotated number of clusters in the data and confirmed the tendency summarized in Fig. 3f. However, the identified partitions are generally composed of well-separated clusters. On the Quake Limb Muscle dataset, *contrastive-sc* + KMeans managed to identify an almost perfect partitioning (ARI 0.99). A similarly good result is achieved with scziDesk (0.97); scDeepCluster managed to create an embedding with well-defined clusters; however, they were in less agreement with the ground truth (0.76 ARI score). This misalignment between the internal and external scores is a general tendency for scDeepCluster, extending to most of the other datasets; it explains the results in Fig. 3c, placing scDeepCluster as the best performing method on Silhouette scores. On the Quake Bladder dataset, scziDesk outperformed *contrastive-sc* + KMeans, which incorrectly split the bladder cell types into two large subgroups. As indicated in the underlying ground truth visualization, this differentiation was produced during the representation learning phase and could be traced back to the input cells' distribution in future works. Furthermore, the Quake Bladder dataset has an extreme class imbalance (containing clusters of sizes 1203, 1167, 73 and 57). In this context, our method divided the largest group, also having the highest sample variance. On the Romanov dataset, *contrastive-sc* + KMeans identified the partitioning closest to the ground truth with an ARI score of 0.73.

**Fig. 8:**
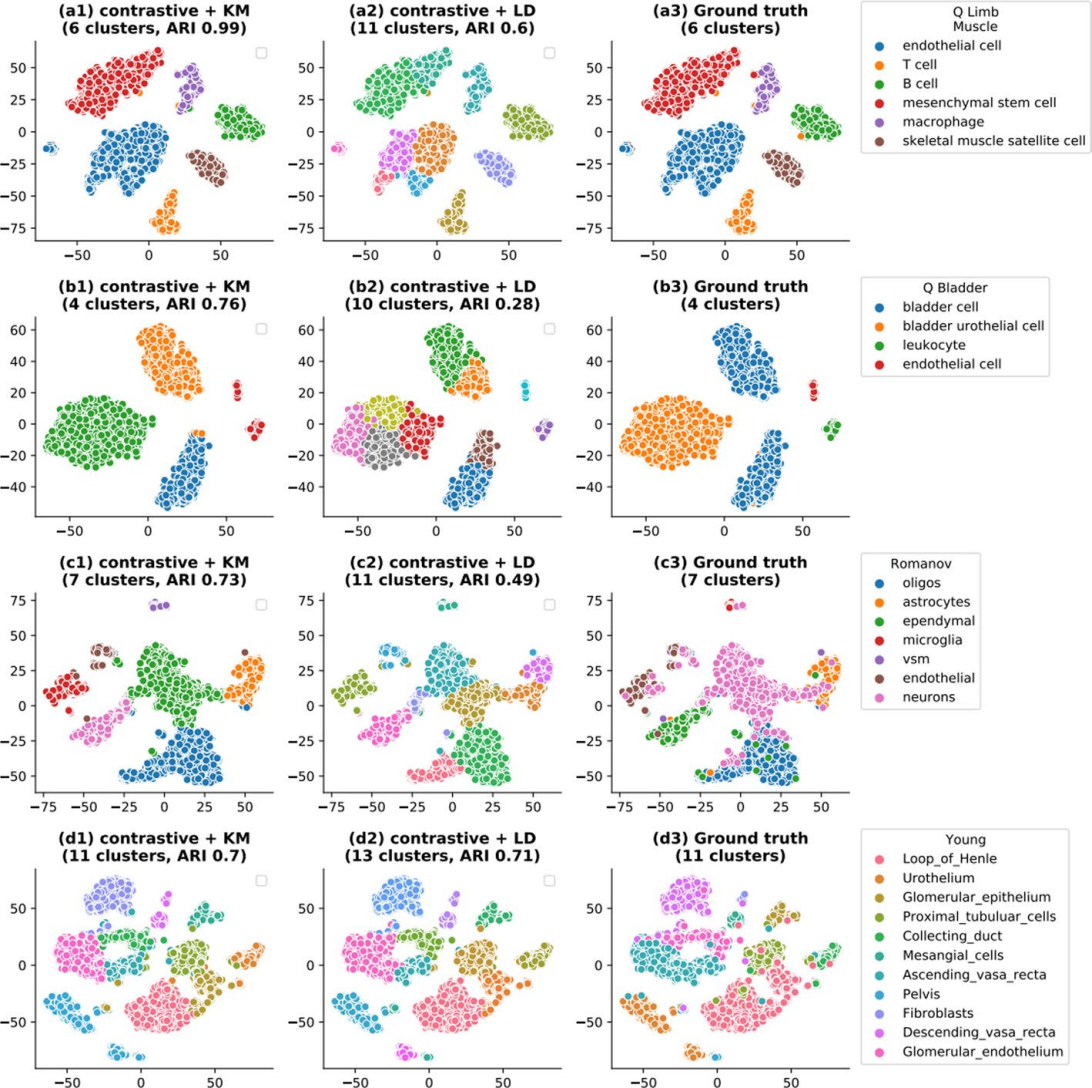
Contrastive-sc clustering compared to ground truth. The comparison of predicted and ground truth clusters on 4 scRNA-seq datasets (Quake Limb Muscle, Muraro, Romanov, Young) is displayed. Our methods (contrastive + KM and contrastive + LD) clustered the same embedding and we also illustrated the ground truth in this space. All plots present a 2D t-SNE projection of the underlying embeddings. contrastive + LD consistently overestimated the number of clusters in the data and performed best on datasets with a large number of clusters

A detailed validation of results has been conducted relative to the ground truth information, depicted in Fig. 8. Leiden identified several partitions within the endothelial and mesenchymal stem cells on the Limb muscle dataset and thus predicted twice as many clusters as in the ground truth annotation. Similar behaviors can be observed on the remaining datasets. Unlike KMeans based methods, the performance of Leiden is relatively better on datasets having a higher number of clusters. Additionally, a positive correlation between the ARI score of *contrastive-sc* + Leiden ARI and the number of clusters in the dataset is observed (Additional file 1: Fig. S5b). However, the identified subclusters have a spatial continuity in the 2D t-SNE projection, which leads us to believe that they may correspond to cellular sub-types. On the Limb Muscle dataset, *contrastive-sc* + KMeans produces a nearly perfect partition (ARI score 0.99), the errors being caused by a few mesenchymal stem cells confounded with macrophage cells as well as a T-cell confounded with a B-cell. A comprehensive analysis of this dataset indicates that 28 cells out of a total of 3909 cells have been assigned to the incorrect cluster, and as illustrated in Additional file 1: Fig. S4, most of the incorrectly predicted cells have low expression values. This visualization exercise demonstrated that most of the embeddings and cell-groups identified with *contrastive-sc* were aligned with the ground truth while forming well-separated clusters, confirming the encouraging average results as ARI and Silhouette scores, reported in Fig. 3a, c.

### Impact of the selected clustering algorithm

Different clustering algorithms may have different behaviors, computational costs and produce different partitions of the same input data. This section explores the specificities of the two selected clustering algorithms (KMeans and Leiden) and compares their performance with a set of another 5 state-of-the-art clustering methods.

Leiden clustering does not require prior knowledge about the expected number of clusters, making it more suitable to be employed in a knowledge discovery setting. Our experiments employed the Leiden implementation integrated into the Scanpy package [14]. The Leiden [27] graph-clustering performs community detection based on optimizing modularity and processes the cells' neighborhood graph. Seurat computes the graph using a PCA representation of the data matrix, while our method uses the learned embedding directly. *contrastive-sc* + KMeans requires knowing the number of input clusters in the data. The same constraint applies to half of the selected methods (i.e. scziDesk, scDeepCluster, scrna, cidr). Unlike KMeans, community detection is not biased towards identifying equal-sized clusters. Leiden community detection is 7 times more computationally expensive than KMeans (Table 3), and this gap increases with the number of input cells. If *contrastive-sc* + KMeans is used in an exploratory setting (i.e. to determine the optimal number of clusters in the data) a computational cost growing linearly with the number of explored values should be foreseen.

*contrastive-sc* computes by default a cell partitioning with KMeans or Leiden. However, the decoupling between the representation learning and the clustering phase allows the flexibility to cluster the produced cell embedding with any other tool. A set of 5 clustering methods (Birch, GMM, MeanShift, Spectral Clustering and Ward Hierarchical Clustering), implemented in the scikit-learn library^3^ have been used to cluster the same embedding created by *contrastive-sc*. The predicted partition has been evaluated using the ARI score (Table 2). The results indicate no best performing method across all datasets, suggesting the data specificities play an essential role in the clustering performance. On average, KMeans and Ward Hierarchical clustering performed best across all datasets and achieved the same mean score (0.77). The NMI scores show that Ward Hierarchical Clustering (Additional file 1: Table S6) is marginally better than KMeans (achieving 0.81 instead of 0.80). The internal scores (Additional file 1: Tables S7, S8) place KMeans as the best performing method. MeanShift [45] determines the number of clusters based on the sample relative density and outperformed Leiden community detection (0.51), achieving an ARI score of 0.65. However, MeanShift was on average 30 times slower than Leiden (Table 3). The execution time analysis indicates that KMeans is the most computationally efficient method, 3 times faster than the next alternatives (e.g. Birch, GMM, Ward Hierarchical Clustering). This exercise demonstrated our method’s flexibility to integrate with other clustering frameworks to search and identify the optimal configuration depending on the analyzed data. Moreover, choosing KMeans and Leiden as default clustering algorithms provided the best trade-off between performance, usage requirements and efficiency.

**Table 2:** Comparison between 7 clustering methods, applied on the embedding learned with *contrastive-sc*

	Dataset name	KMeans	Leiden^a^	Birch	GMM	MeanShift^a^	Spectral Clustering	Hierarchical Clustering
1	Quake Smart seq2 Trachea	0.86	0.3	0.87	0.88	0.78	0.84	**0.89**
2	Quake10 × Bladder	**0.75**	0.29	**0.75**	**0.75**	0.72	**0.75**	**0.75**
3	Quake10 × Spleen	**0.91**	0.17	0.72	0.74	0.84	0.83	0.9
4	Quake Smart seq2 Diaphragm	**0.98**	0.7	**0.98**	**0.98**	0.96	**0.98**	**0.98**
5	Quake10 × Limb Muscle	**0.99**	0.58	0.9	0.91	0.69	0.96	**0.99**
6	Quake Smart seq2 Limb Muscle	**0.98**	0.67	0.97	0.92	0.92	0.97	**0.98**
7	Romanov	**0.73**	0.49	0.52	0.48	0.62	**0.73**	0.69
8	Adam	0.83	0.67	0.64	0.61	0.54	**0.84**	0.8
9	Muraro	0.91	0.57	0.85	0.85	0.83	0.85	**0.92**
10	Young	0.66	**0.68**	0.57	0.6	0.39	0.65	0.65
11	Quake Smart seq2 Lung	**0.59**	0.49	**0.59**	0.49	0.48	0.57	0.6
12	10 PBMC	**0.7**	0.51	0.61	0.6	0.66	0.66	0.69
13	Mouse ES cells	0.73	0.52	0.64	0.64	0.69	0.72	**0.74**
14	Worm neuron cell	**0.49**	**0.49**	0.34	0.36	0.15	0.47	**0.49**
15	Mouse bladder cell	0.46	**0.56**	0.53	0.5	0.51	0.42	0.44
	**Average score**	**0.77**	0.51	0.70	0.69	0.65	0.75	**0.77**

The results depict the average ARI score across 3 consecutive runs. The methods annotated with ^a^are those where the correct number of clusters was not provided as input. The complete analysis of the remaining clustering scores has been provided in Additional file 1: Tables S6–S8

The best scores per dataset are highlighted in bold

**Table 3:** Average execution time of clustering algorithms

	KMeans	Leiden^a^	Birch	GMM	MeanShift^a^	Spectral Clustering	Hierarchical Clustering
Avg execution time	**0.14**	7.94	0.44	0.46	14.01	0.78	0.48
Speed factor relative to KMeans	**1**	56.71	3.17	3.29	100.09	5.60	3.47

We measured the mean duration across 3 consecutive runs on clustering the embedding produced by *contrastive-sc* for all real-world datasets. The methods annotated with ^a^are those where the correct number of clusters was not provided as input. KMeans provides the highest computational speed from the set of 7 explored clustering algorithms

The fastest methods are highlighted in bold

### Method stability

**Fig. 9:**
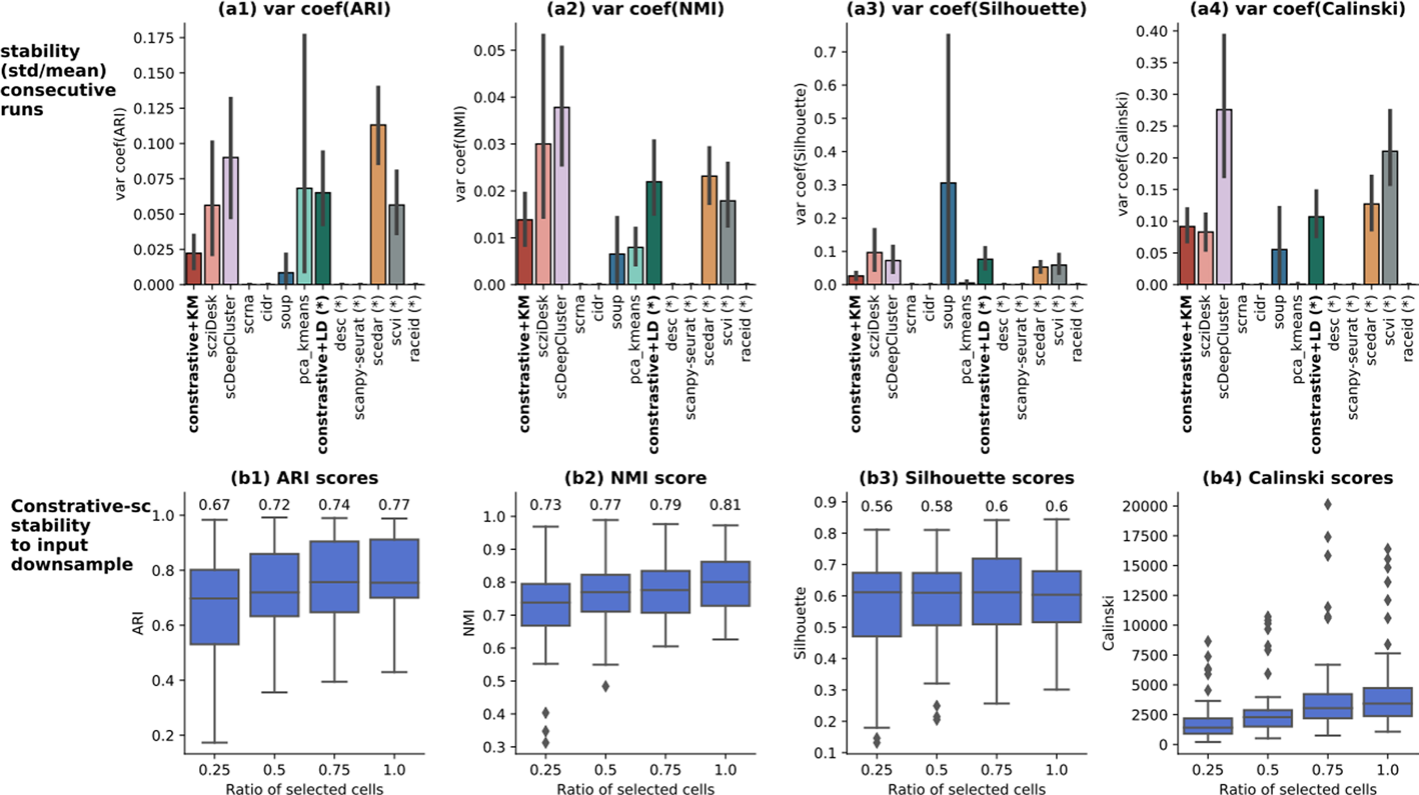
Model stability across consecutive runs (**a1**–**a4**) and to input downsample (**b1**–**b4**). The model stability across 3 runs on the real-world datasets has been depicted as the coefficient of variation (for each dataset, the standard deviation across runs divided by the average result). Here, the lower the score, the more stable the model. For this experiment, the input data consisted of all input cells in each dataset. The second analysis assesses how the model performance changes when only a fraction of cells is passed as input. Three different stratified subsets have been generated randomly for each dataset, selecting 25%, 50%, 75% and 100% of all cells in each of the benchmarked real datasets. The annotated values represent the mean score of each experiment. The performance remains relatively stable: providing half of the dataset reduces the ARI score by 5, while only 25% of data attains an ARI score of 0.67

The stability of our method across runs and regarding input downsampling has been assessed. First, the stability across consecutive runs has been evaluated using the coefficient of variation (the standard deviation divided by the mean score) for each computed clustering score (Fig. 9a1–a4). The coefficient of variation normalizes the variance in the data by the mean, which makes it a suitable method to compare results across datasets having a diverse range of scores. *contrastive-sc* displays an average stability on all evaluators. The closest competitor methods, scziDesk and scDeepCluster are generally more unstable. *contrastive-sc* + Leiden produces more variant partitions than *contrastive-sc* with KMeans. Given that the 2 clustering algorithms (KMeans and Leiden) processed the same embedding, the clustering algorithm was responsible for the variations in the predicted partition. The most unstable methods were scedar and scDeepCluster on external evaluators. On internal evaluators, soup, scDeepCluster and scvi had the highest variability across consecutive runs.

The stability to input downsample analysis was performed by assessing the change in method performance when only partial input data was provided. Our experimental setup consisted of evaluating the results when only 25%, 50%, 75% and 100% of cells were provided as input. The sampling has been performed in a random and stratified way, to ensure a similar representation of all clusters across experiments. For each setting, three random samples have been generated. The results depicted in Fig. 9b1–b4 show that selecting half of the input cells reduces slightly the ARI score and the Silhouette, while only 25% of input cells are still able to provide competitive results (0.67 ARI and 0.56 Silhouette). These experiments indicate that *contrastive-sc* is robust to input downsampling.

### Analysis of data augmentation techniques

**Fig. 10:**
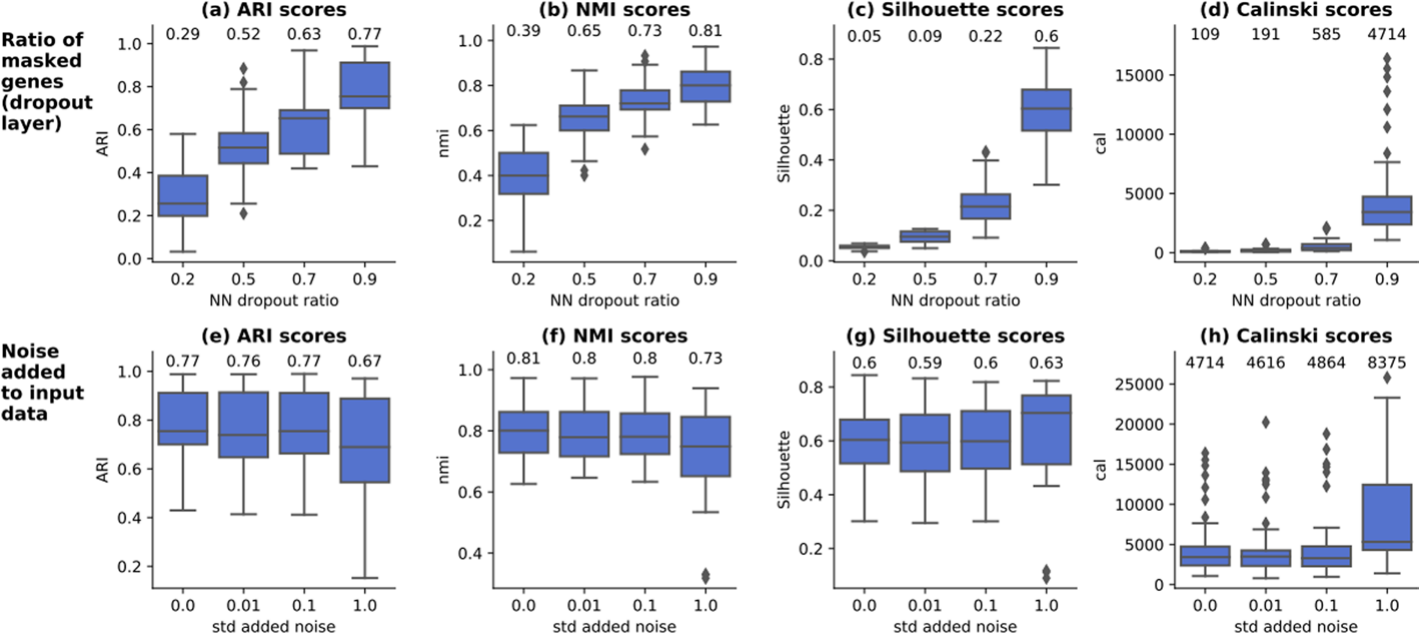
Analysis of data augmentation techniques for neural network dropout (**a**–**d**) and input noise (**e**–**h**). Models masking a different ratio (0.2–0.9) of input genes through dropout NN layers have been trained on all real-world datasets. Both internal and external evaluators indicate that the performance increases with the NN dropout rate and peaks at the dropout rate = 0.9. Gaussian random noise has been added to the input data having a standard deviation from 0.01 to 1. Our results indicate that the addition of noise does not improve model performance. The annotated values represent the mean across all underlying experiments. For each setting 3 consecutive runs have been performed

The representation learning phase relies on data augmentation techniques to create variations in input samples, as required by the contrastive loss. Two data augmentation techniques have been analyzed in detail: the addition of **dropout** and the addition of **noise** to input data. For disambiguation, this dropout does not refer to the false zero count events occurring on scRNA-seq data but to a type of layer in artificial neural networks removing (dropping out) an amount of neuron connections to the forward layer. When applied directly on the input data (gene expression per cell), dropout masks an arbitrary ratio of randomly selected genes (by giving a weight of 0 to connection in the neural-network). The augmented views consist only of a small fraction of the input data that are different every epoch. This strategy allows the model to learn in detail about various input genes instead of focusing on a few important features. An experimental setting consisting of applying dropout ratios from 0.2 to 0.9 across all real datasets has been implemented, as depicted in Fig. 10a–d. The results indicate that our method’s performance grows with the dropout ratio on external and internal scores and peaks at 0.9 dropout ratio. This finding is aligned with the claims in image analysis [21], that strong data augmentation techniques bring a gain in performance for contrastive representation learning. The strong NN dropout can be seen as the equivalent of image cropping typically employed on computer vision tasks, which consists of creating the augmented copies as random segments (i.e. crops) of the original image. When analyzing the cat image depicted in Fig. 1, selecting only the rectangle around the ears or around the eyes are examples of random crops, transformations that select only a subset of the original image and hide the remaining parts. It was shown [46] that such transformations work successfully because they create occlusion invariance, giving the model the ability to recognize the content even when a fraction of the input data is being provided. On scRNA-seq data, strong NN dropout allows the model to learn about the importance of each individual gene and minimize the impact of the context (the expression of all other genes) which could mask relevant gene-level details. The higher the dropout, the more gene-level granularity is achieved. contrastive-sc analyzes only the most variable genes, which also have a lower sparsity level than the overall dataset. When the number of input genes is 1000, a dropout of 90% maintains 100 genes, which appear to be enough to represent correctly the cell. However, the results would also deteriorate as the dropout approaches 100%, in which case the risk of selecting one or only a few genes contaminated by dropout (or irrelevant for the cell identity) increases. This corresponds to selecting only one pixel as representative of the cat image.

The second explored image augmentation technique consisted of adding gaussian random noise to the input data. The experimental results depicted in Fig. 10e–h indicate that this strategy does not improve the performance. As the input datasets have a significant sparsity level, on average above 85% (Additional file 1: Table S4), adding noise to all input genes, even with a reduced standard deviation, would incorrectly alter the sparsity corresponding to the genes not expressed in a given cell. Thus, the network is encouraged to learn biologically incorrect gene-cell relationships, which justifies performance degradation.

### Network architecture search

**Fig. 11:**
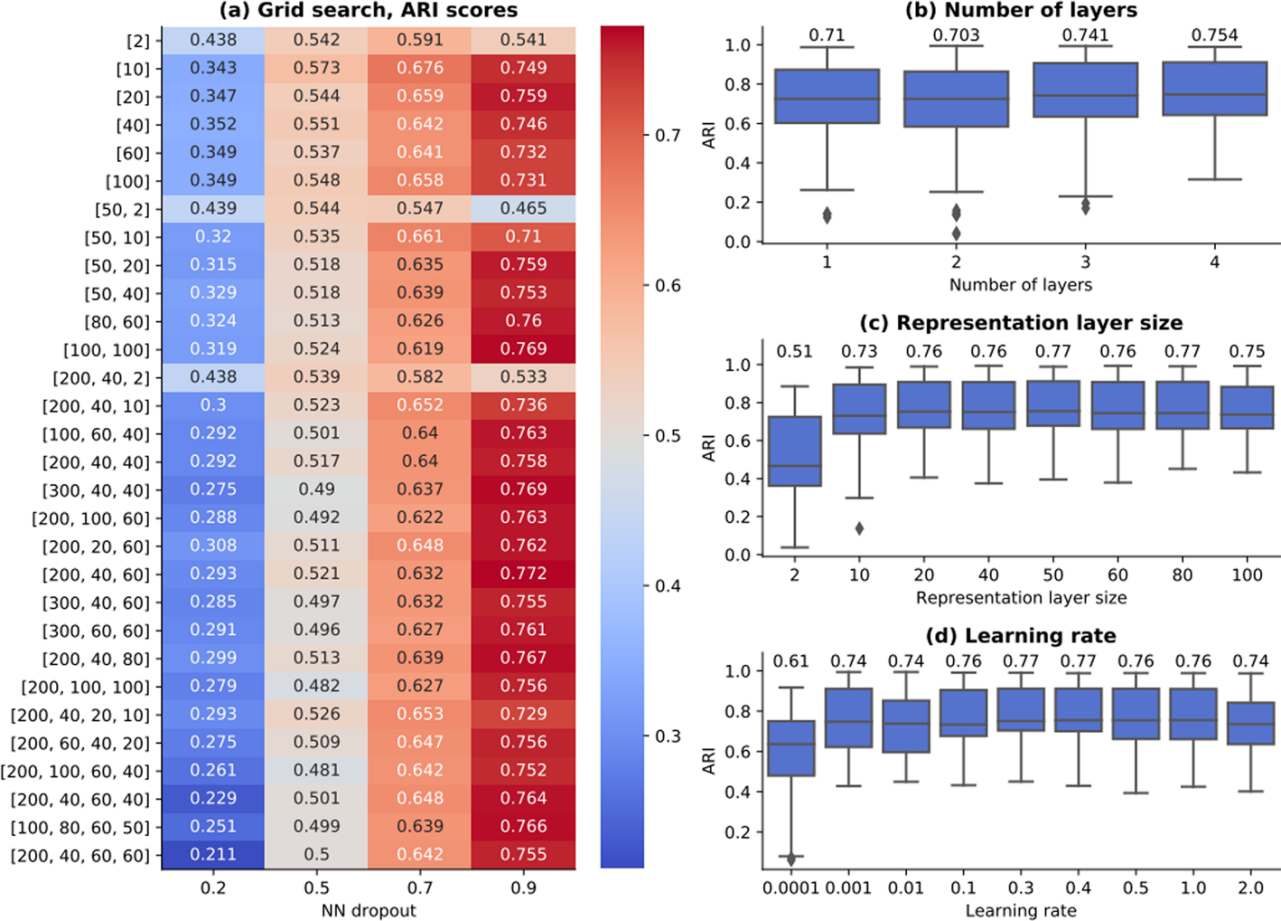
Network architecture search. A set of 30 neural network architectures consisting of 1–4 stacked linear layers have been trained on all real datasets (**a**). The labels indicate the network layer sizes (i.e. [60, 20] is a network composed of 2 linear layers of size 60 and 20, the latter also representing the cell embedding size). All values in **a**–**d** represent ARI scores of 3 runs for each experiment. The network dropout is the only parameter having a significant impact on model performance. The number of layers (**b**) influence only marginally the model output. Similar results have been obtained for the embedding size (**c**), as long as it is large enough (> 10 values). Various learning rates from 0.0001 to 2 have been explored (**d**) and indicate that the optimal value is 0.4. The annotated values represent the mean across all underlying experiments

The performance of predictive models depends on the set of parameters defining the model architecture (i.e. the number of NN layers, their sizes) and the training phase (i.e. the optimizer, the learning rate). Usually, these parameters are strongly dependent on the input dataset. An experimental study has been conducted to identify the optimal architecture for *contrastive-sc* (the encoder model) on all real-work datasets and study the model’s sensitivity to variations in input configurations. As illustrated in Fig. 11a, a set of 30 network structures have been generated, consisting of from 1 to 4 stacked linear layers, each of size from 2 to 200. Across all network architectures, the neural network dropout is the only parameter having a significant impact on model performance. Figure 11b indicates that the network depth influences only marginally the model performance. The same is valid for the embedding layer size (Fig. 11c), as long as it is large enough (> 10). Even when using a single layer of 2 values, the proposed training method can learn a sample separatrix, as shown by the average ARI score of 0.49.

The model training was performed with an Adam optimizer, an initial learning rate of 0.4 and a cosine scheduler, as proposed in [21]. The learning rate has been validated with a grid search, exploring values from 0.0001 to 2. The results depicted in Fig. 11d indicate that in addition to being the optimal learning rate for contrastive learning on scRNA-seq data, the model performance is stable when sampling other learning rates in the neighborhood of 0.4. All performed experiments indicate that the model performance is stable across an extended range of network architectures and optimization settings.

## Discussion

In this section we review the overall advantages and the limitations of contrastive-sc while comparing it with the other explored methods. The encouraging experimental results summarized in Figs. 2 and 3 show that self-supervised contrastive learning constitutes a good alternative to the analytical way of modeling the dropout in order to acquire robustness for clustering scRNA-seq data, using NB or ZINB autoencoders [15, 16, 19]. Even though there is no one method performing best across all datasets, *contrastive-sc* compares favorably on external and internal scores with the best performing techniques on simulated (scedar, scziDesk, scanpy-seurat) and real datasets (scziDesk, scDeepCluster, desc, scanpy-seurat). The selection of input genes combined with the quick training time places *contrastive-sc* among the most computationally efficient state-of-the-art clustering methods; moreover, it scales well with an increasing number of cells or genes in the input data. The gain in execution time allows extending the range of exploratory experiments to perform neural network architecture search or input parameter optimization. The decoupling between the representation learning phase and clustering provides the flexibility to employ other clustering algorithms easily or integrate the learned embeddings in other methods. Thus, both scenarios of data exploration and data exploitation can be addressed.

The extensive experimental study conducted on the clustering methods for scRNA-seq data allows us to perform a meta-analysis highlighting the idiosyncrasies of each method and also to place *contrastive-sc* in the landscape of state-of-the-art clustering models.

### Methods meta-analysis

The collected experimental data allowed us to perform methods comparison and meta-analysis. The correlations between the internal and the external clustering measures have been explored in Additional file 1: Fig. S5a. As expected, ARI scores are significantly correlated with the other external quality measure, the NMI score. The correlation with the internal measures varies significantly from one method to the other. Weaker values indicate that when the identified partition is in agreement with the ground truth, it has a low cluster separability and conversely. *contrastive-sc* exhibits a strong positive correlation with the Silhouette scores, both when using KMeans and Leiden community detection (Pearson correlation coefficient 0.77 and respectively 0.66), indicating that the high ARI scores also correspond to well-defined clusters. Calinski scores are not bounded, and their scale depends on the dataset specifics (number of samples/clusters); as such, their relevance should be assessed only in relative terms and excluded from the correlation analysis.

The results depicted in Fig. 4 indicated that no method performs best across all datasets. In an attempt to identify method idiosyncrasies, we explored the relationship between dataset specificities and model performance. Each dataset is characterized by its number of clusters, the sparsity ratio, the mean/median/max values, the skew and the kurtosis. The Pearson correlation values between these properties and all method scores on real datasets are illustrated in Additional file 1: Fig. S5b. Some methods are impacted negatively by a higher data sparsity (i.e. scDeepCluster, scrna). As detailed in Additional file 1: Table S4, the maximum count values vary in orders of magnitude from one dataset to another, ranging from 219 to 1.242.300. Some methods provide competitive results on datasets having larger count values (e.g. scedar) while others conversely (e.g. scrna). A similar observation can be made about skew and kurtosis, associated with a degradation in the performance of scrna, and on the naive baseline.

*contrastive-sc* works best on datasets with fewer clusters when using the KMeans clustering and conversely for Leiden. This phenomenon can be explained by the documented tendency KMeans has to identify equal-sized [46], combined with the significant class imbalance associated with the datasets having more than 8 clusters (Table 1).

## Future works

For the representation learning training phase, Chen et al. [23] recommended using larger batch sizes (i.e. 4096 samples). However, most of the studied scRNA-seq datasets have less than 4000 samples. In our experiments, we employed a fixed batch size of 200 samples. We foresee exploring in future works more recent and improved approaches to representation learning, leveraging techniques providing state-of-the-art results with smaller batch sizes, such as the Moco frameworks [47, 48].

Following the first set of experiments presented in this publication, we foresee several possible optimizations. One promising idea is to leverage the cluster assignment predictions of the representation embedding as pseudo-labels and formulate the clustering problem as a classification task under label noise [49]. Another research track is to enrich the encoder model with a clustering layer, as proposed in scziDesk, which would allow the network to perform the task of cluster assignment. A topic studied only in a few publications [50] is the early stopping condition for representation learning. Most publications [23, 48, 51] propose a dataset- related number of epochs and do not elaborate on the evolution of performance or overfitting in the absence of an annotated dataset. We foresee an investigation in terms of the stability of learned representations which could be used as an early stopping condition. Last but not least, enriching the current method with a model interpretability step could help to explain the model’s behavior and provide insights into the features (i.e. genes) responsible for the cluster assignments of individual cells.

## Conclusions

This paper introduced *contrastive-sc*, a new method leveraging contrastive self-supervised learning for clustering scRNA-seq data. We conducted an extensive experimental study to illustrate the importance of the data augmentation strategy and gene selection, to identify the optimal neural network architecture. Our results showed that *contrastive-sc* is stable across runs, robust to input downsample, relatively insensitive to changes in input configuration parameters, computationally efficient both as execution time and memory footprint and scalable to larger datasets.

The main contributions can be summarized as follows:

- Adaptation of contrastive self-supervised training proposed for image processing to scRNA-seq data.
- Analysis of the proposed method on a range of 24 simulated and 15 real-world datasets, supported by a detailed ablation study. The proposed method showed encouraging results across all analyzed datasets, which were achieved in a computationally efficient way.
- Comparative analysis of the landscape of state-of-the-art clustering methods for scRNA-seq data.

Our results showed that there is no constant best-performing method across all analyzed datasets. However, *contrastive-sc* compared favorably with other state-of-the-art methods and achieved top scores on average, both on external and internal clustering metrics. We hope that the current work inspires future research in contrastive learning and provides a bridge between other recent achievements in parallel research tracks such as computer vision and bioinformatics.

## Supplementary Information

**Additional file 1.** Supplementary method (Contrastive loss); Supplementary Tables S1 to S8; Supplementary Figures S1 to S5.

## Data Availability

All data needed to reproduce the presented results has been made available on GitHub (https://github.com/ciortanmadalina/contrastive-sc).

## Abbreviations

D: Input dataset
n: Number of observations
d: Number of dimensions (features)
NN: Artificial neural network
ARI: Adjusted Rand Index
NMI: Normalized Mutual Information
KL: Kullback–Leibler

## Footnotes

1 https://scikit-learn.org/stable/modules/generated/sklearn.cluster.KMeans.html.

2 https://github.com/vtraag/leidenalg.

3 https://scikit-learn.org/stable/modules/clustering.html.
