# Supplementary material for: Contrastive self-supervised clustering of scRNA-seq data
Source: BMC Bioinformatics. 2021 May 27;22:280. doi: 10.1186/s12859-021-04210-8 (PMC8157426; doi:10.1186/s12859-021-04210-8)
Supplement: Supplementary file 1 — Additional file 1. Supplementary method (Contrastive loss); Supplementary Tables S1 to S8; Supplementary Figures S1 to S5. [file 12859_2021_4210_MOESM1_ESM.pdf]

Contrastive self-supervised clustering of scRNA-seq data

Supplementary Materials

Madalina Ciortan, Matthieu Defrance

Interuniversity Institute of Bioinformatics in Brussels

Université Libre de Bruxelles, Belgium

Corresponding author: matthieu.dc.defrance@ulb.ac.be

Contrastive loss

The contrastive loss proposed in [1] is applied on this final layer in order to guide the model to map similar views to neighboring representations and dissimilar ones to non-neighboring ones. Given a minibatch with N samples, the two sample views are concatenated and create a structure indexed by $1 \leqslant i \leqslant 2N$, where $pair(i)$ is the batch index of the pair for sample i, z_i is the embedding of x_i. The loss takes the form:

$L^{contrastive} = \sum_{i=1}^{2N} L_i^{contrastive}$, with

$$L_i^{contrastive} = -log \frac{exp\left(z_i \cdot z_{pair(i)}/\tau\right)}{\sum_{i=1}^{2N} 1_{i \neq j}\, exp\left(z_i \cdot z_{pair(i)}/\tau\right)}$$

where $pair(i)$ is the batch index of the pair for sample i, z_i is the embedding of x_i, $z_i \cdot z_{pair(i)}$ represents the dot product between the normalized embedding vectors, τ is a temperature parameter set in our experiments to the recommended value of 0.07, $1_B \in \{0,1\}$ is the indicator function returning 1 iff B evaluates to true, or explicitly $1_{i \neq j} = 1\ if\ i \neq j$ and $1_{i \neq j} = 0\ if\ i = j$.

	Method	Requires number of clusters	Programming language	Availability
1	PCA + K-Means	yes	Python	https://scikit-learn.org/stable/modules/generated/sklearn.decomposition.PCA.html<br><br>https://scikit-learn.org/stable/modules/generated/sklearn.cluster.KMeans.html
2	scDeepClustering	yes	Python	https://github.com/ttgump/scDeepCluster
3	scziDesk	yes	Python	https://github.com/xuebaliang/scziDesk
4	scanpy/Seurat	no	Python	https://scanpy.readthedocs.io/en/stable/tutorials.html#clustering
5	desc	no	Python	https://github.com/eleozzr/desc
6	scRNA	yes	Python	https://github.com/nicococo/scRNA
7	scedar	no	Python	https://scedar.readthedocs.io/en/latest/index.html
8	cidr	yes	R	https://github.com/VCCRI/CIDR
9	soup	yes	R	https://rdrr.io/github/lingxuez/SOUP/
10	scvi	no	R	https://github.com/YosefLab/scvi-tools
11	raceid	no	R	https://cran.r-project.org/web/packages/RaceID/vignettes/RaceID.html

Table S1. Accessibility and programming language of benchmarked methods.

	Dataset Name	Size (cells x genes)	Sparsity	Max value	Mean	Median	Skew	Kurtosis
1	data_-1c4	1000 x 2500	27.90%	2675	34	10	6	49
2	data_0c4	1000 x 2500	34.23%	2675	36	12	6	45
3	data_1.5c4	1000 x 2500	50.77%	2675	45	15	5	34
4	data_1c4	1000 x 2500	44.39%	2675	41	14	5	38
5	data_1c8	2000 x 2500	46.88%	2282	43	12	5	27
6	data_-1c8	2000 x 2500	29.72%	2282	34	9	5	36
7	data_0c8	2000 x 2500	36.35%	2282	37	10	5	33
8	data_1.5c8	2000 x 2500	53.39%	2282	48	13	4	24
9	data_0c16	4000 x 2500	34.43%	2516	36	11	6	46
10	data_1.5c16	4000 x 2500	51.38%	2516	46	14	5	35
11	data_-1c16	4000 x 2500	27.92%	2516	34	10	6	51
12	data_1c16	4000 x 2500	44.84%	2516	42	13	5	39

Table S2. Descriptive statistics of balanced simulated dataset. All statistics (mean, median, skew, kurtosis) have been computed only on the non-zero values.

	Dataset Name	Size (cells x genes)	Sparsity	Max value	Mean	Median	Skew	Kurtosis
1	data_-1c4	3000 x 2500	28.68%	3019	34	10	7	73
2	data_0c4	3000 x 2500	35.24%	3019	37	11	7	67
3	data_1.5c4	3000 x 2500	52.20%	3019	46	14	6	51
4	data_1c4	3000 x 2500	45.68%	3019	42	13	6	57
5	data_1c8	3000 x 2500	45.62%	3333	42	13	6	57
6	data_-1c8	3000 x 2500	28.64%	3333	34	10	7	74
7	data_0c8	3000 x 2500	35.19%	3333	37	11	7	68
8	data_1.5c8	3000 x 2500	52.11%	3333	46	14	6	51
9	data_0c16	3000 x 2500	35.19%	3134	37	11	7	66
10	data_1.5c16	3000 x 2500	52.14%	3134	46	14	6	50
11	data_-1c16	3000 x 2500	28.63%	3134	34	10	7	72
12	data_1c16	3000 x 2500	45.63%	3134	42	13	6	56

Table S3. Descriptive statistics of imbalanced simulated dataset. All statistics (mean, median, skew, kurtosis) have been computed only on the non-zero values.

	Dataset Name	Sparsity	Max value	Mean	Median	Skew	Kurtosis
1	Quake Smart seq2 Trachea	85.48%	678254	219	62	68	6354
2	Quake10x Bladder	86.94%	2959	5	1	17	686
3	Quake10x Spleen	94.34%	2324	3	1	25	2431
4	Quake Smart seq2 Diaphragm	91.35%	208892	249	45	46	2849
5	Quake10x Limb Muscle	93.57%	1259	3	1	23	992
6	Quake Smart seq2 Limb Muscle	89.47%	1242300	316	78	102	18782
7	Romanov	85.92%	8642	3	1	94	19080
8	Adam	92.33%	4929	3	1	89	12820
9	Muraro	73.02%	1597	4	2	43	2304
10	Young	94.70%	4666	4	1	45	4004
11	Quake Smart seq2 Lung	89.08%	822470	320	65	82	10691
12	10X PBMC	92,23%	777	3.24	1	15	460
13	Mouse ES Cell	65,76%	2309	2.42	1	25	4949
14	Worm neuron cell	98,61%	219	1.76	1	18	970
15	Mouse Bladder cell	94,86 %	303	2.06	1	16	525

Table S4. Descriptive statistics of single-cell datasets. All statistics (mean, median, skew, kurtosis) have been computed only on the non-zero values.

	Dataset Name	Optimal input size	Silhouette score	AIC
1	Quake Smart seq2 Trachea	500	0.61	-779753
2	Quake10x Bladder	500	0.81	-1533450
3	Quake10x Spleen	500	0.70	-5948416
4	Quake Smart seq2 Diaphragm	1000	0.84	-502236
5	Quake10x Limb Muscle	500	0.62	-2209972
6	Quake Smart seq2 Limb Muscle	500	0.80	-629835
7	Romanov	500	0.53	-1712599
8	Adam	500	0.51	-2018976
9	Muraro	500	0.65	-1248460
10	Young	1000	0.46	-2994687
11	Quake Smart seq2 Lung	1000	0.60	-920851
12	10X PBMC	500	0.56	-2580395
13	Mouse ES Cell	1000	0.61	-1535567
14	Worm neuron cell	5000	0.32	-2021032
15	Mouse Bladder cell	1500	0.36	-1586925

Table S5. Optimal number of most variable genes per dataset, selecting by the underlying Silhouette scores, averaged over 3 runs for each experiment. The corresponding Silhouette and AIC scores have been presented in the last 2 columns.

	Dataset Name	Kmeans	Leiden (*)	Birch	GMM	Mean Shift (*)	Spectral Clustering	Ward Hierarchical Clustering
1	Quake Smart seq2 Trachea	0.84	0.6	0.82	0.84	0.72	0.79	**0.87**
2	Quake10x Bladder	0.8	0.57	0.79	0.8	0.77	0.8	**0.81**
3	Quake10x Spleen	0.79	0.47	0.69	0.71	0.74	0.72	**0.84**
4	Quake Smart seq2 Diaphragm	**0.96**	0.85	**0.96**	**0.96**	0.93	0.95	**0.96**
5	Quake10x Limb Muscle	0.97	0.81	0.94	0.94	0.79	0.94	**0.98**
6	Quake Smart seq2 Limb Muscle	**0.96**	0.85	0.94	0.92	0.9	0.95	0.**96**
7	Romanov	**0.72**	0.63	0.59	0.58	0.64	0.71	0.68
8	Adam	0.85	0.79	0.75	0.73	0.71	0.85	0.82
9	Muraro	0.86	0.77	0.83	0.83	0.81	0.82	0.87
10	Young	0.78	0.8	0.72	0.74	0.64	0.76	0.77
11	Quake Smart seq2 Lung	0.77	0.76	0.77	0.74	0.74	0.76	0.77
12	10 PBMC	0.73	0.67	0.69	0.69	0.73	0.71	0.73
13	Mouse ES cells	0.71	0.68	0.67	0.67	0.61	0.69	0.75
14	Worm neuron cell	0.63	0.65	0.55	0.55	0.49	0.61	0.64
15	Mouse bladder cell	0.7	0.72	0.7	0.69	0.71	0.66	0.69
	**Average score**	0.80	0.71	0.76	0.76	0.73	0.78	**0.81**

Table S6. Comparison between 7 clustering methods, applied on the embedding learned with contrastive-sc. The results depict the average NMI score across 3 consecutive runs. The methods annotated with (*) indicate those where the correct number of clusters has not been provided as input.

	Dataset Name	Kmeans	Leiden (*)	Birch	GMM	Mean Shift (*)	Spectral Clustering	Ward Hierarchical Clustering
1	Quake Smart seq2 Trachea	0.61	0.38	0.58	0.57	0.38	0.6	0.59
2	Quake10x Bladder	0.81	0.28	0.8	0.8	0.7	0.81	0.8
3	Quake10x Spleen	0.7	0.21	0.47	0.51	0.47	0.65	0.69
4	Quake Smart seq2 Diaphragm	0.84	0.49	0.84	0.84	0.69	0.83	0.83
5	Quake10x Limb Muscle	0.62	0.48	0.6	0.59	0.28	0.61	0.61
6	Quake Smart seq2 Limb Muscle	0.8	0.52	0.79	0.73	0.54	0.8	0.8
7	Romanov	0.53	0.41	0.39	0.34	0.39	0.53	0.51
8	Adam	0.51	0.4	0.42	0.41	0.24	0.5	0.48
9	Muraro	0.65	0.44	0.59	0.58	0.37	0.62	0.66
10	Young	0.46	0.46	0.34	0.36	0.18	0.43	0.43
11	Quake Smart seq2 Lung	0.6	0.58	0.52	0.52	0.39	0.57	0.6
12	10 PBMC	0.56	0.35	0.48	0.51	0.55	0.52	0.52
13	Mouse ES cells	0.61	0.27	0.48	0.49	0.41	0.59	0.59
14	Worm neuron cell	0.32	0.3	0.2	0.21	0.04	0.3	0.3
15	Mouse bladder cell	0.36	0.39	0.28	0.28	0.31	0.32	0.33
	**Average Score**	**0.60**	0.40	0.52	0.52	0.40	0.58	0.58

Table S7. Comparison between 7 clustering methods, applied on the embedding learned with contrastive-sc. The results depict the average Silhouette score across 3 consecutive runs. The methods annotated with (*) indicate those where the correct number of clusters has not been provided as input.

	Dataset Name	Kmeans	Leiden (*)	Birch	GMM	Mean Shift (*)	Spectral Clustering	Ward Hierarchical Clustering
1	Quake Smart seq2 Trachea	1118.05	1350.01	1031.58	1018.51	443.96	1071.66	1061.94
2	Quake10x Bladder	7433.61	8414.36	7121.08	7343.4	3835.85	7413.45	7322
3	Quake10x Spleen	12509.95	8166.01	7920.46	8604.58	2679.72	10896.19	11350.73
4	Quake Smart seq2 Diaphragm	5075.86	4261.39	5066.02	5065.46	2528.83	4939.54	4573.64
5	Quake10x Limb Muscle	3360.11	3684.95	3158.18	2957.01	793.63	3240.69	3291.62
6	Quake Smart seq2 Limb Muscle	4059.13	3789.95	3703.38	3594.93	1325.07	3984.51	3894.46
7	Romanov	2805.26	2511.01	1495.07	1522.04	631.15	2709.36	2373.46
8	Adam	2343.25	2035.34	1706.8	1534.43	304.43	2288.52	2056
9	Muraro	3118.36	2858.11	2696.22	2479.54	848.82	2564.68	3017.35
10	Young	3032.7	2708.99	1886.43	2075.11	229.35	2704.98	2721.43
11	Quake Smart seq2 Lung	1454.1	1796.95	1293.09	1275.04	651.33	1328.88	1424.96
12	10 PBMC	15187.7	11983.11	10833.4	9480.23	3904.49	12120.96	13331.2
13	Mouse ES cells	3925.63	2505.63	2103.28	2172.3	363.01	3494.37	3560.69
14	Worm neuron cell	1188.78	1077.38	753.05	781.23	30.03	1097.86	1049.82
15	Mouse bladder cell	4105.6	4449.99	2825.41	2835.4	763.63	2280.47	3774.22
	**Average score**	**4714**	4106	3572	3515	1288	4142	4320

Table S8. Comparison between 7 clustering methods, applied on the embedding learned with contrastive-sc. The results depict the average Calinski score across 3 consecutive runs. The methods annotated with (*) indicate those where the correct number of clusters has not been provided as input.

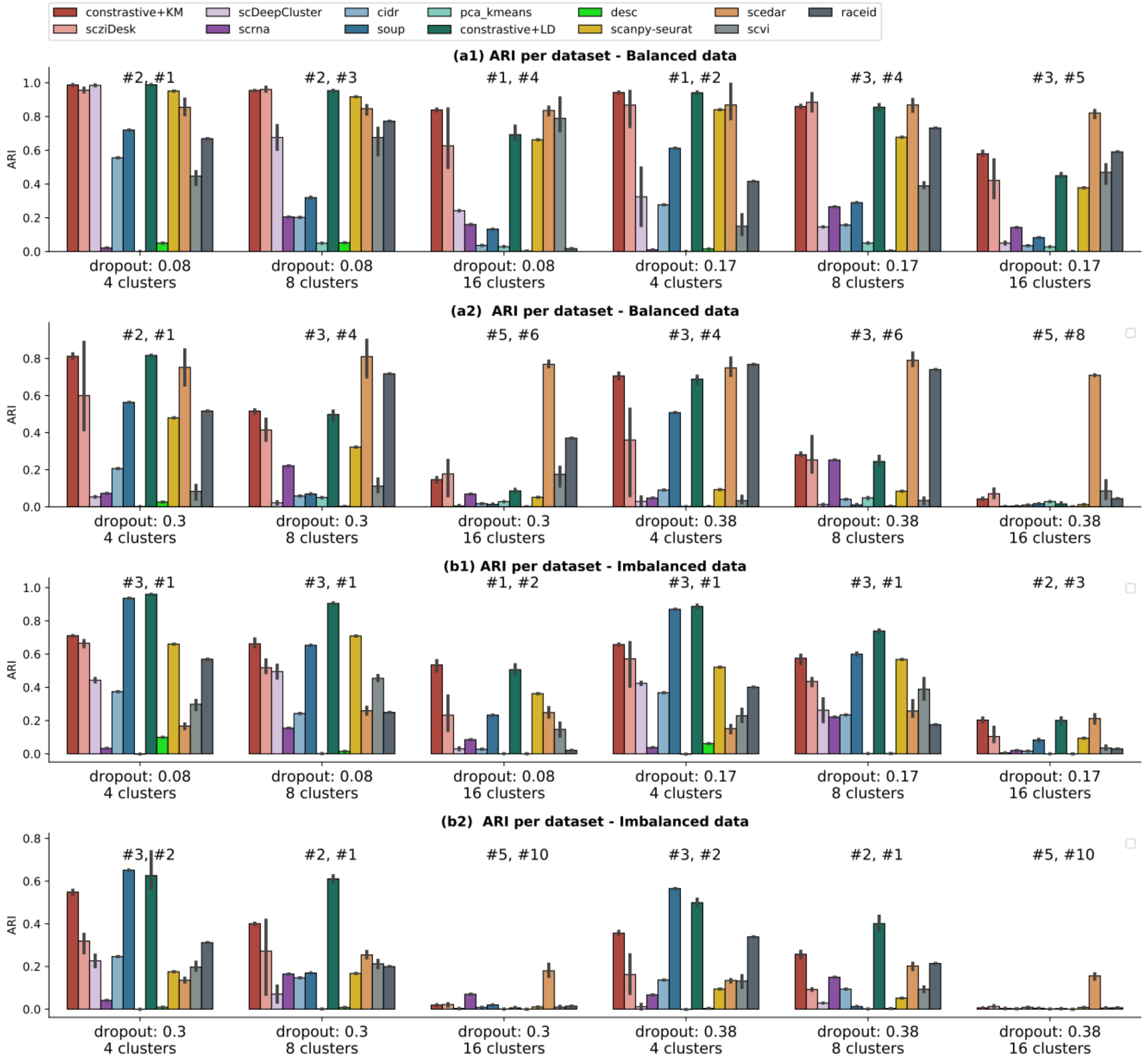

Fig S1. Dataset ranking by ARI score for simulated data balanced (panel a1, a2) and imbalanced (panel b1, b2). We studied 4 levels of dropout rate: 0.08 (a), 0.16 (b), 0.3 (c) and 0.38 (d). The level of dropout is reported by the simulation library and is lower than the data sparsity. For each setting 4, 8 and 16 clusters have been generated. The presented results assess the ARI score over 3 runs for each dataset using different initialization seeds. A dataset level analysis has been depicted in panels a and b, where the method ranking of contrastive+KMeans and contrastive+Leiden has been depicted as # rank number, representing the position our scores had within the 13 explored techniques.

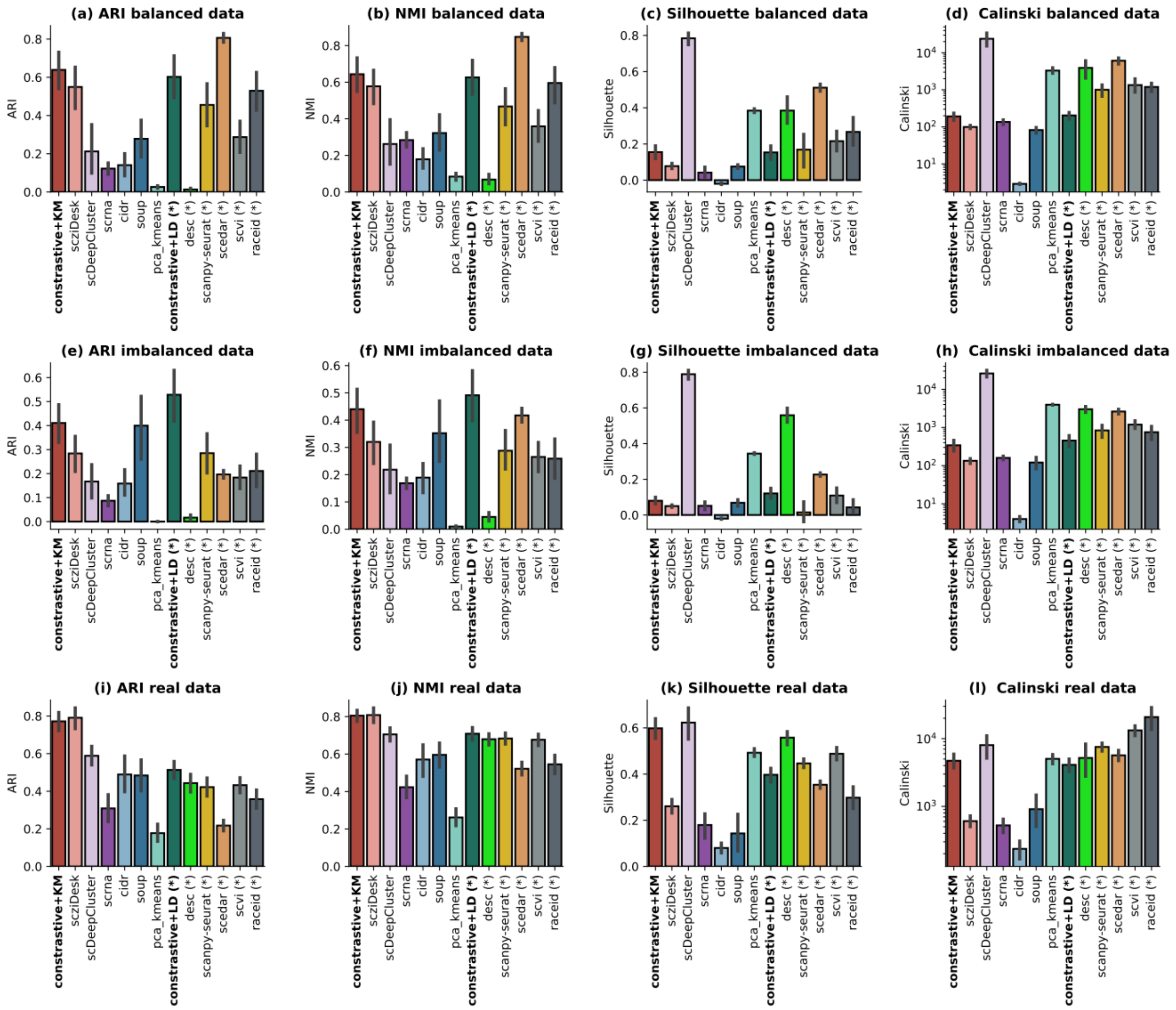

Fig S2. Overview of all explored datasets (simulated balanced, imbalanced and real world) using all selected scores (ARI, NMI, Silhouette, Calinski).

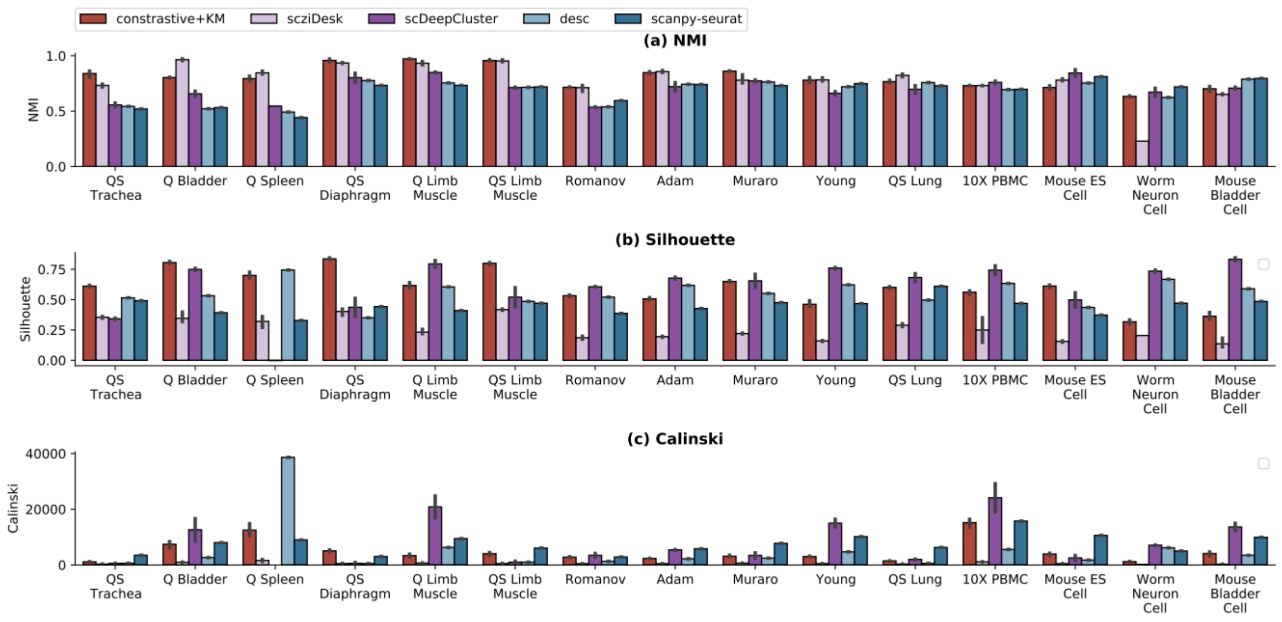

Fig S3. Method results on biological datasets in terms of NMI, Silhouette and Calinski scores. For a compact display, we selected the top 5 best performing methods. The results aggregate 3 consecutive runs of all 13 explored methods over the 15 biological datasets. For simplicity, only top 5 performing methods have been selected.

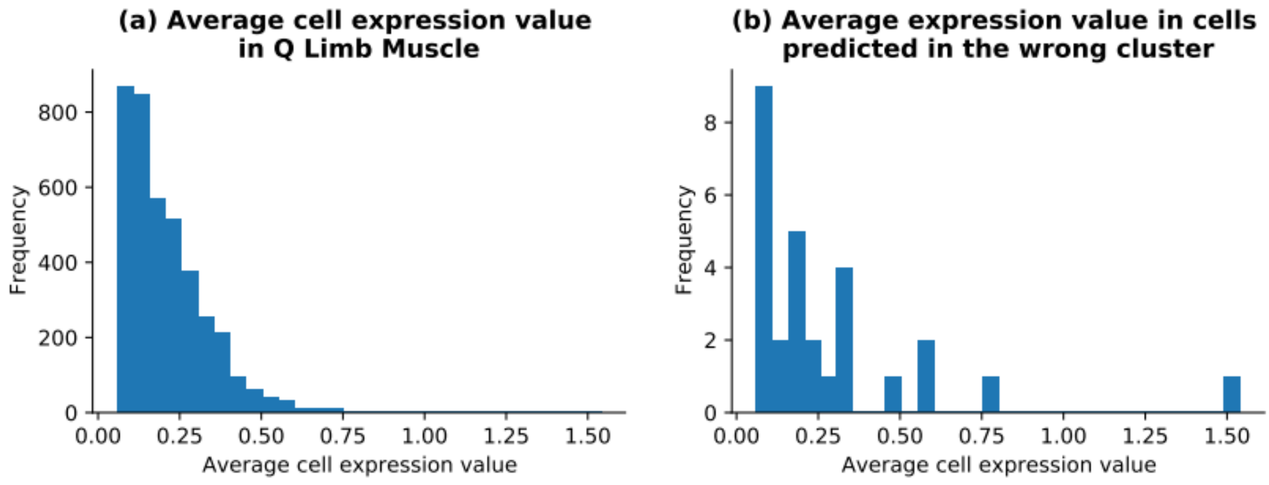

Fig S4. Distribution of average cell expression values across all cells in Q Limb Muscle dataset (panel a) compared to the cells predicted by contrastive+Kmeans in the wrong cluster. Most of the incorrect predictions have been made on cells having low expression values.

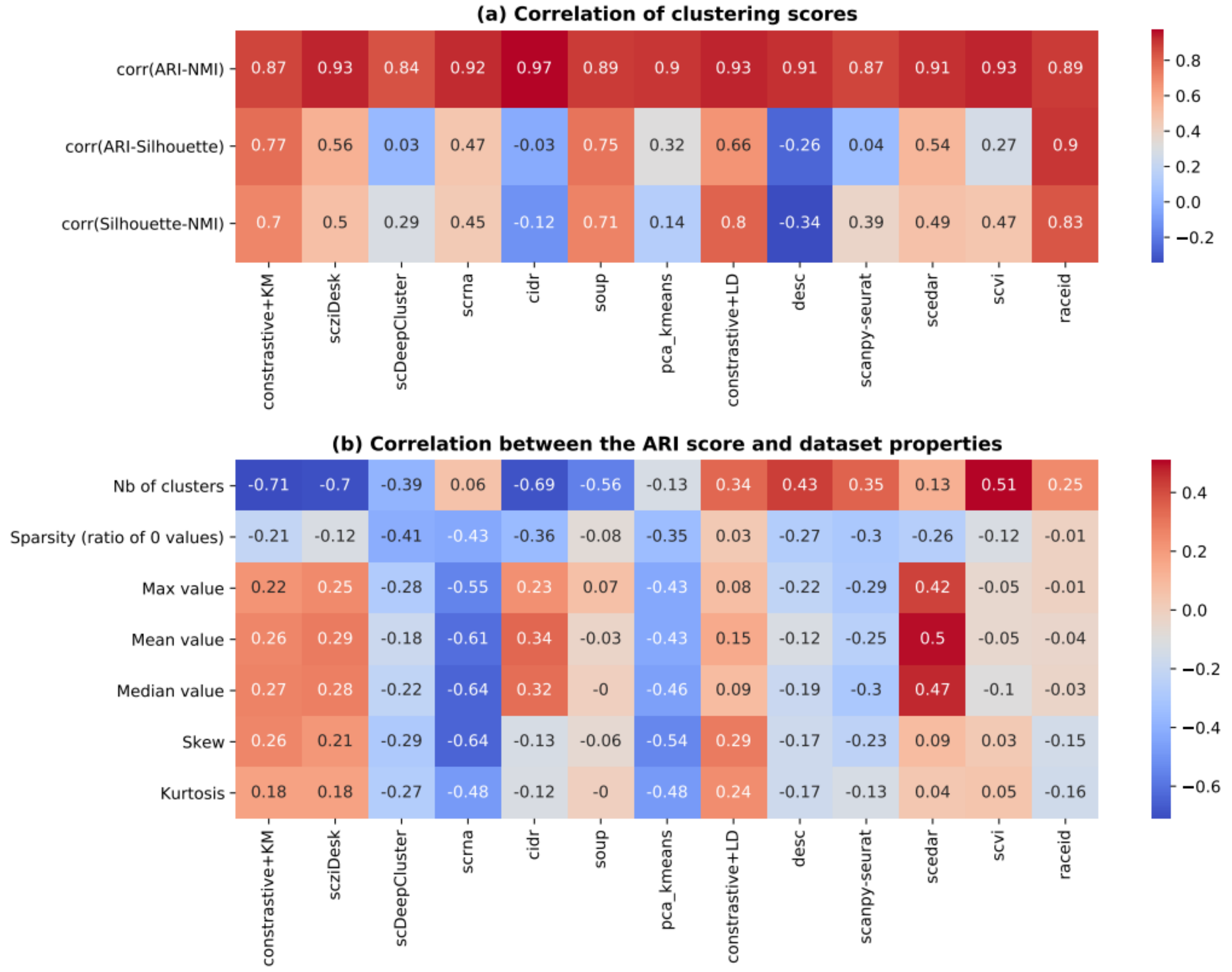

Fig S5. **Meta-analysis of explored methods** The Pearson correlation between ARI, Silhouette scores and the remaining measures has been illustrated in panel a. The correlation is computed for each method, by comparing the ARI, Silhouette and NMI scores obtained after 3 runs on all real-world datasets. The ARI score is significantly correlated with NMI across all experiments. However, the internal scores are not always aligned with external measures. Weaker correlations with internal quality measures indicate that identified clusters are well separated but do not match the ground truth and conversely. The Pearson correlation between dataset specificities and method performance as ARI score has been depicted in panel b. The input data consisted of the ARI scores reported on all real-world scRNA-seq datasets and each dataset's properties. For each dataset, we explored the number of clusters, the sparsity ratio, the mean/median/max values, the skew and the kurtosis. Some methods (contrastive+KMeans, scziDesk, cider) work best on a reduced number of clusters, while others are impacted negatively by data sparsity (e.g. scDeepCluster, scrna).
